# Long-Term Application of Bio-Compost Increased Soil Microbial Community Diversity and Altered Its Composition and Network

**DOI:** 10.3390/microorganisms10020462

**Published:** 2022-02-17

**Authors:** Xiayan Liu, Yu Shi, Lingyu Kong, Lihong Tong, Haoxuan Cao, Hu Zhou, Yizhong Lv

**Affiliations:** 1 Department of Soil and Water Sciences, College of Land Science and Technology, China Agricultural University, Beijing 100193, China; liuxiayan@cau.edu.cn (X.L.); 13427569158@163.com (L.K.); caohaoxuan21@163.com (H.C.); 2 State Key Laboratory of Crop Stress Adaptation and Improvement, School of Life Sciences, Henan University, Kaifeng 475004, China; yshi@henu.edu.cn; 3 State Key Laboratory of Hydroscience and Engineering, Department of Hydraulic Engineering, Tsinghua University, Beijing 100084, China; tonglihong@mail.tsinghua.edu.cn

**Keywords:** bio-compost, microbial alpha-diversity, taxonomic biomarkers, co-occurrence network, microbial function

## Abstract

The influence of bio-compost on the diversity, composition and structure of soil microbial communities is less understood. Here, Illumina MiSeq sequencing and a network analysis were used to comprehensively characterize the effects of 25 years of bio-compost application on the microbial diversity of soil and community composition. High dosages of bio-compost significantly increased the bacterial and fungal richness. The compositions of bacterial and fungal communities were significantly altered by bio-compost addition. Bio-compost addition enriched the relative abundance of beneficial microorganisms (such as *Sphingomonas*, *Acidibacter*, *Nocardioides*, etc.) and reduced the relative abundance of harmful microorganisms (such as *Stachybotrys* and *Aspergillus*). Electrical conductivity, soil organic matter and total phosphorus were the key factors in shaping soil microbial community composition. The bacterial network was more complex than fungal network, and bacteria were more sensitive to changes in environmental factors than fungi. Positive interactions dominated both the bacterial and fungal networks, with stronger positive interactions found in the bacterial network. Functional prediction suggested that bio-composts altered the soil bacterial-community metabolic function with respect to carbon, nitrogen and phosphorus cycles and fungal community trophic modes. In conclusion, suitable bio-compost addition is beneficial to the improvement of soil health and crop quality and therefore the sustainability of agriculture.

## 1. Introduction

The application of fertilizers can significantly change the nutrient availability of plants and the diversity and function of microorganisms [1]. The excessive use of chemical fertilizer has caused a series of environmental problems (such as the decrease in nutrient utilization efficiency, soil quality deterioration, etc.) [2]. Soil microorganisms not only have extremely rich genetic and functional diversity, but also play important roles in soil nutrient conversion, maintaining soil productivity, and promoting the sustainable development of ecosystems [3]. Soil microbial-community diversity and composition are also important indicators that reflect the evolution of soil quality. Studies have shown that long-term application of chemical fertilizers significantly decreased the bacterial richness of soil and its diversity, and disturbed the ecological balance of soil microbial communities [4,5]. Organic amendments have been shown to be more effective than inorganic fertilizer in improving soil biological characteristics [6], especially in terms of increasing the microorganisms beneficial to plant growth, and decreasing plant pathogenic microorganisms [7]. Thus, an effective way to solve the problems caused by the excessive application of chemical fertilizers is to apply organic amendments alone or in combination with inorganic fertilizers [8,9,10]. 

Compost is an important form of organic amendment, which plays an important role in improving soil properties and crop growth [11,12]. Some researchers found that compost addition could improve soil microbial activity and diversity [9,13]. However, some other studies have demonstrated that compost has neutral or negative effects on the soil microbial community, which may be due to differences in compost type, dosage and duration time [14,15,16]. Therefore, it is necessary to use long-term experiments to study the effects of compost on soil microbial diversity and community structure. Bio-compost is a type of compost that is compounded by microorganisms with specific functions using organic waste (such as animal manure, straw and sewage sludge) [12]. Bio-compost is considered to be more effective than normal compost because it is rich in beneficial microbial flora and physiologically active substances (such as indoleacetic acid, gibberellin, vitamins and amino acids). However, there is no consistent conclusion that has been reached about the effects of bio-compost on soil microbial diversity and community composition, especially the microbial function. 

Network analysis is used to explore the microbial interactions between different microbial taxa under different fertilization treatments [17]. In addition, we can also find key functional microorganisms that have an important influence on the microbial community structure and potential functions by network analysis, which helps to deeply understand the diversity and function of the microbial community [18]. 

To sum up, it is of great significance to carry out research on the effects of bio-compost on the composition, structure and function of soil bacterial and fungal communities so as to elucidate the internal mechanism of soil microbial changes after the application of bio-compost, and the promotion of soil ecological health. Therefore, based on a 25-year long-term field experiment, we systematically studied the effect of long-term application of bio-compost on soil microbial composition, co-occurrence and function using high-throughput sequencing of soil bacteria 16S rRNA gene and fungal ITS gene data, network analysis, and Phylogenetic Investigation of Communities by Reconstruction of Unobserved States (PICRUSt) and FUNGuild. The objectives of this study were to (i) study the differences in soil microbial diversity, composition and community structure under long-term different fertilization treatments; (ii) analyze the correlation between soil properties and microbial communities under different fertilization treatments; (iii) analyze bacterial and fungal co-occurrence patterns and predict the function of bacteria and fungi; (iv) preliminarily reveal the response mechanism of soil microorganisms to long-term different fertilization. We hypothesized that the application of bio-compost would significantly improve soil microbial diversity, increase beneficial microorganisms, decrease disease-causing microorganisms, and alter soil microbial co-occurrence patterns and metabolic functions.

## 2. Materials and Methods

### 2.1. Experimental Site and Design

This field experiment began in 1996 at Quzhou Experimental Station of China Agricultural University in Quzhou County, Hebei Province, China (115°02′ E, 36°87′ N). The soil is classified as inceptisols soil (American Soil Taxonomy). The planting system is the rotation of winter wheat and corn. This region has a sub-humid warm temperate continental monsoon climate [19], and the annual mean temperature and precipitation are 13.1 ℃ and 556.2 mm.

There are four treatments in this study, specifically,: no fertilizer addition (CK), chemical fertilizer addition (CF), 15,000 kg ha^−1^ compost addition (high dosage of bio-compost, EMI), and 7500 kg ha^−1^ compost addition (conventional dosage of bio-compost, EMII). Each treatment was replicated three times; therefore, we had a total of 12 plots (8.0 m × 4.0 m) following a randomized block design. Ammonium bicarbonate (18% N), Urea (46% N) and superphosphate (16% P_2_O_5_) were used as chemical fertilizer at a rate of 1125 kg ha^−1^, 600 kg ha^−1^ and 1125 kg ha^−1^, respectively, for each crop season. Bio-compost was made by mixing straw, chicken manure, wheat bran and cottonseed meal (dry weight: 6:3:0.5:0.5), then, by spraying 2‰ (volume/weight) effective microorganism (EM)agent solution on the raw-material pile evenly, mixing and composting according to EM micro ecological engineering technology. The EM agent, provided by Beijing Wotu Tiandi Biotechnology Co., Ltd., and its microbial composition and contents are shown in Table 1. All the amendments were evenly distributed on the surface of the plot, and then rotated into the soil. The soil background information before the start of the experiment is shown in Table S1.

### 2.2. Soil Sampling

Soil samples were collected in October 2019, one week before harvesting maize. The samples were collected from the surface layer (0–20 cm) at seven random locations per plot using a soil auger, and then were wrapped in ice packs and transported to the laboratory. The sample of each plot was divided into two parts, one part was used to determine the physicochemical properties of soil and the other part for DNA extraction.

### 2.3. Analysis Methods

#### 2.3.1. Analysis of Soil Basic Properties

The water content of the soil was determined by the oven-drying method [20]. Soil pH and electrical conductivity (EC) were analyzed using the pH meter (PHS-3D, Shanghai, China) and the conductivity meter (DDS-11A, Shanghai, China), respectively, under water suspension (1:5 *w*/*v*). Soil organic matter (SOM) was measured by the dichromate digestion method. Available potassium (AK) was extracted with 1 mol L^−1^ NH_4_OAc and determined by a flame emission spectrophotometer (FP640, INASA, Shanghai, China). Available phosphorus (AP) was extracted with 0.5 mol L^−1^ NaHCO_3_, which was measured using the molybdenum blue method. Total phosphorus (TP), total nitrogen (TN), ammonium (NH_4_^+^-N) and nitrate (NO_3_^−^-N) were extracted with H_2_SO_4_-HClO_4_, H_2_SO_4_ and 1mol L^−1^ KCl, respectively, which were determined with a continuous flow analyzer (Autoanalyer 3, SEAL, Norderstedt, Germany). 

#### 2.3.2. Soil DNA Extraction and High-Throughput Sequencing Analysis

Soil DNA was extracted using Power Soil DNA Isolation Kit (MoBio Laboratories, Carlsbad, CA, USA) according to the manual. The V3-V4 hypervariable gene regions of the bacterial 16S rRNA and the fungal ITS gene regions were subjected to high-throughput sequencing by Beijing Allwegene Tech, Ltd. (Beijing, China), and amplified with the primers as shown in Table S2 [21,22]. 

For bacteria, the PCR was carried out on a Mastercycler Gradient (Eppendorf, Germany) using 25 μL reaction volumes, containing 12.5 μL 2xTaq Plus Master Mix, 1 μL Forward Primer (5 µM), 1 μL Reverse Primer (5 µM), 3 μL BSA(2 ng μL^−1^), and 7.5 μL ddH2O. Cycling parameters were 94 °C for 5 min, followed by 28 cycles of 94 °C for 30 s, 55 °C for 30 s and 72 °C for 60 s with a final extension at 72 °C for 7 min. The PCR products were purified using an Agencourt AMPure XP Kit. For fungi, we used the same PCR reaction mixture system and sequencing preparation as described above for bacteria. Thermocycling consisted of an initial denaturation at 94 °C for 5 min, followed by 34 cycles of 94 °C for 30 s, 55 °C for 30 s, 72 °C for 45 s and a final extension at 72 °C for 7 min. 

Deep sequencing was performed on Miseq platform at Allwegene Company (Beijing, China). After the run, an image analysis, base calling and error estimation were performed using Illumina Analysis Pipeline Version 2.6. 

#### 2.3.3. Statistics Analysis

The raw sequence reads were initially trimmed using Mothur. The software package Vsearch was used to further filter out sequences that were erroneous and chimeric. The sequences were clustered into operational taxonomic units (OTUs) at a similarity level of 97%, to generate rarefaction curves and to calculate the richness and diversity indices. Bacterial and fungal sequences were deposited in the Sequence Read Archive (SRA) data of National Center for Biotechnology Information under accession numbers SRP336958 and SRP336961, respectively. The Ribosomal Database Project (RDP) Classifier tool was used to classify all sequences into different taxonomic groups. Principal component analysis (PCA) and the analysis of similarities (ANOSIM) were used to examine the similarity between different samples. In order to determine the relationships between soil properties and microbial communities, a Redundancy analysis (RDA), Spearman’s correlation analysis and Mantel test were conducted using the Canoco 5 trial version, SPSS software, version 20 (IBM Corp., Armonk, NY, USA) and R software (Version 4.1.1), respectively. The LEfSe method was conducted to identify potential microbial markers of different fertilization treatments [23]. A PICRUSt analysis was used to predict bacterial functions [24]. The fungal functions were annotated according to the FUNGuild database [25]. A network analysis was conducted to explore the microbial co-occurrence patterns using R software (Version 4.1.1). SPSS software, version 20 (IBM Corp., Armonk, NY, USA) was used to complete statistical analysis. Differences in soil microbial alpha-diversity and the relative abundances of different taxonomic levels of microbe among all treatments were identified by analysis of variance (ANOVA/Duncan). 

## 3. Results

### 3.1. Alpha-Diversity of Bacteria and Fungi 

**Figure 1:**
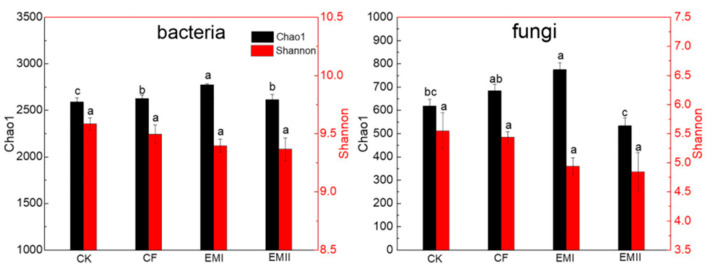
Alpha-diversity of soils bacteria and fungi under different fertilization treatments. Values are means for triplicate replicates. Different lowercase letters on the histogram of the same color indicate significant differences at *p* < 0.05 based on the analysis of variance.

The alpha-diversity indices of soil bacteria and fungi under different treatments are shown in Figure 1. For bacteria, the chao1 indices of EMI and EMII treatments were significantly higher than that of CK treatment by 7.11% and 0.91%, respectively, and the chao1 index of EMI treatment was 5.71% higher than that of CF treatment (*p* < 0.05). Bio-compost treatments decreased the Shannon index, but the difference was not statistically significant. For fungi, compared with CK and CF treatments, the Chao1 index of EMI treatment increased by 25.29% and 13.19%, and the Chao1 index of EMII treatment decreased significantly. The effect of bio-compost on fungal Shannon index was similar as that on bacteria. These results suggested that a high dosage of bio-compost had a greater impact on soil microbial alpha-diversity than CF and EMII treatments.

### 3.2. Soil Bacterial and Fungal Community Structure and Composition

#### 3.2.1. Soil Bacterial and Fungal Community Structure

**Figure 2:**
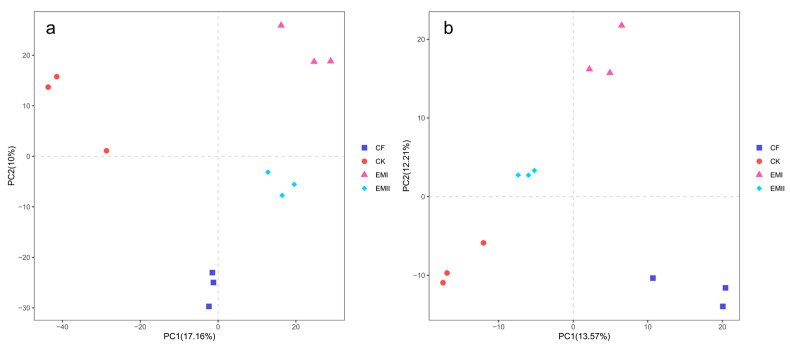
PCA analysis of soil bacteria (**a**) and fungi (**b**) under different fertilization treatments.

The ANOSIM results (bacteria: R = 0.3796, *p* < 0.05; fungi: R = 0.5710, *p* < 0.01) indicated that soil bacterial and fungal communities were significantly different as affected by the bio-compost treatments. It was obvious from the PCA that distinct clustering for different fertilization treatments was observed for bacterial and fungal communities (Figure 2). 

#### 3.2.2. Soil Bacterial and Fungal Community Composition

**Figure 3:**
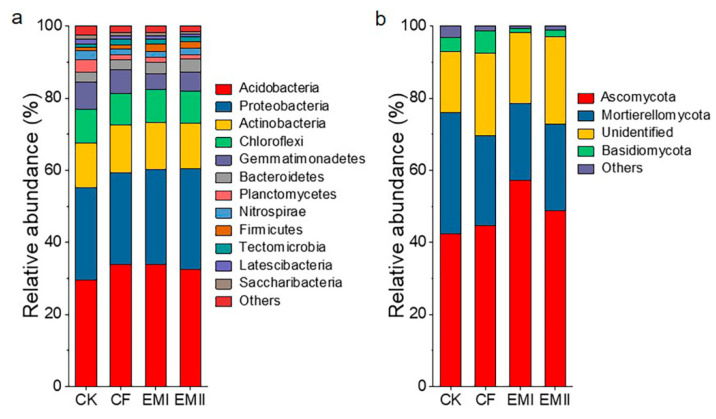
The classification of soil bacterial (**a**) and fungal (**b**) communities at different phylum levels under different fertilization treatments and the relative abundance of each phylum.

A total of 392311 high-quality sequences were obtained in the bacterial community analysis, which were assigned to 39 phyla and 331 genera. Acidobacteria, Proteobacteria, Actinobacteria, Chloroflexi and Gemmatimonadetes were the dominant bacterial phyla across all treatments, accounting for 97.42–98.35% of the total abundance (Figure 3a). Compared with CK, the bio-compost treatments increased the relative abundance of Acidobacteria, Proteobacteria, Actinobacteria, Bacteroidetes and Firmicutes, and decreased that of Chloroflexi, Gemmatimonadetes, Planctomycetes and Nitrospirae. Bio-compost treatments decreased the relative abundance of Acidobacteria relative to CF. The relative abundance of Gemmatimonadetes and Planctomycetes of bio-compost treatments was significantly lower than that of CF and CK treatments (Table S3). 

For fungal communities, we obtained 1561170 high-quality sequences, which were assigned to 13 phyla and 284 genera. Ascomycota (42.34–57.15%), Mortierellomycota (21.28–33.74%), and Basidiomycota (1.33–6.25%) were dominant phyla in all samples (Figure 3b). Compared with CK and CF treatments, bio-compost treatments increased the relative abundance of Ascomycota, and decreased the relative abundance of Mortierellomycota and Basidiomycota (Table S4).

**Figure 4:**
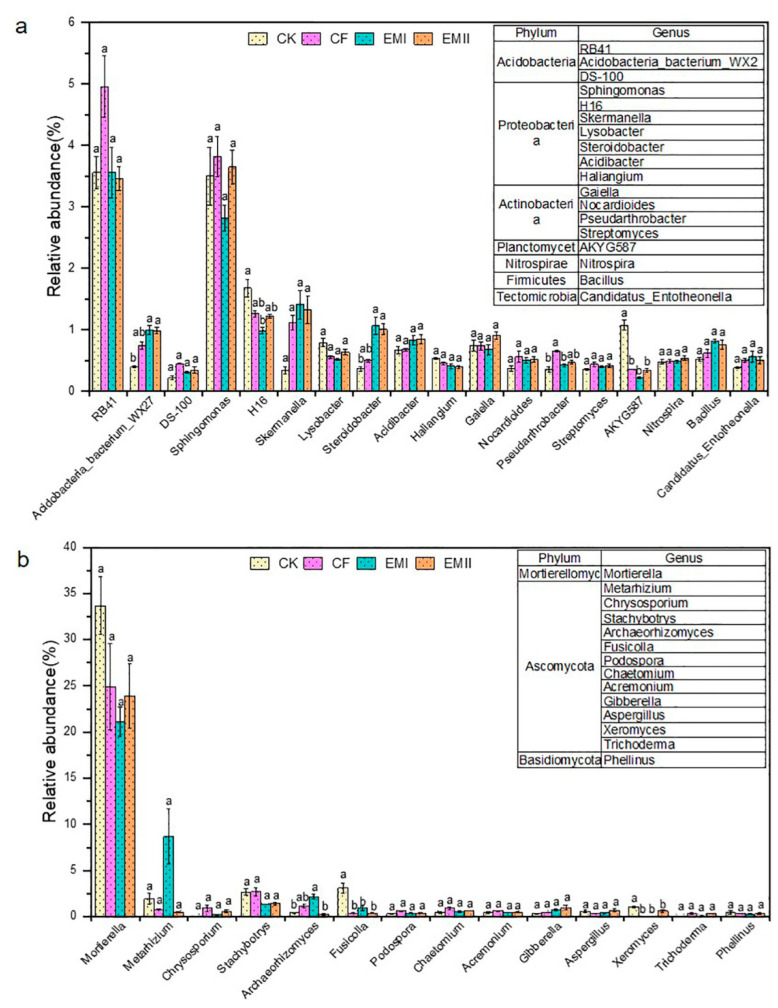
The relative abundance of soil bacterial (**a**) and fungal (**b**) genera under different fertilization treatments. Different letters indicate significant differences under different fertilization treatments (*p* < 0.05).

The bacterial and fungal genera are detailed in Figure 4. *RB41* within Acidobacteria was the dominant bacterial genus of all treatments, whose relative abundance under EMI and EMII treatments was separately 28.22% and 30.24% lower than that of CF (Figure 4a). The relative abundance of *Acidobacteria-bacterium-WX27* in EMI and EMII treatments was, respectively, 149.66% and 148.33% significantly higher than that of CK. In addition, Proteobacteria was composed of *Sphingomonas*, *Desulfurellaceae-H16*, *Skermanella*, etc. *Sphingomonas* (2.82–3.82%) was the dominant bacterial genus of all treatments, for which the relative abundance under EMII treatment was higher than that of CK treatment. Compared with CK, bio-compost treatments increased the relative abundance of *Skermanella*, *Steroidobacter*, and *Acidibacter*, and decreased the relative abundance of *H16* and *Lysobacter*. The application of the bio-compost increased the relative abundance of many beneficial genera (such as *Bacillus*, *Nitrospira*, *Nocardioides*, *Pseudarthrobacter* and *Streptomyces*).

Regarding fungal genera, *Mortierella* (21.14–33.67%) was the most abundant fungal genus in all treatments, and the bio-compost treatments decreased its relative abundance relative to CK and CF treatments (Figure 4b). Most genera belonged to Ascomycota. Compared with CK, bio-composts increased the relative abundance of *Chrysosporium*, *Podospora*, *Chaetomium*, *Acremonium*, *Gibberella* and *Trichoderma*, and decreased that of *Fusicolla*, *Xeromyces* and *Stachybotrys*. Compared with CK and CF treatments, high dosage of bio-compost increased the relative abundance of *Metarhizium* and *Archaeorhizomyces*, and decreased that of *Aspergillus*. 

#### 3.2.3. Taxonomic Biomarkers of Soil Microbial Communities

**Figure 5:**
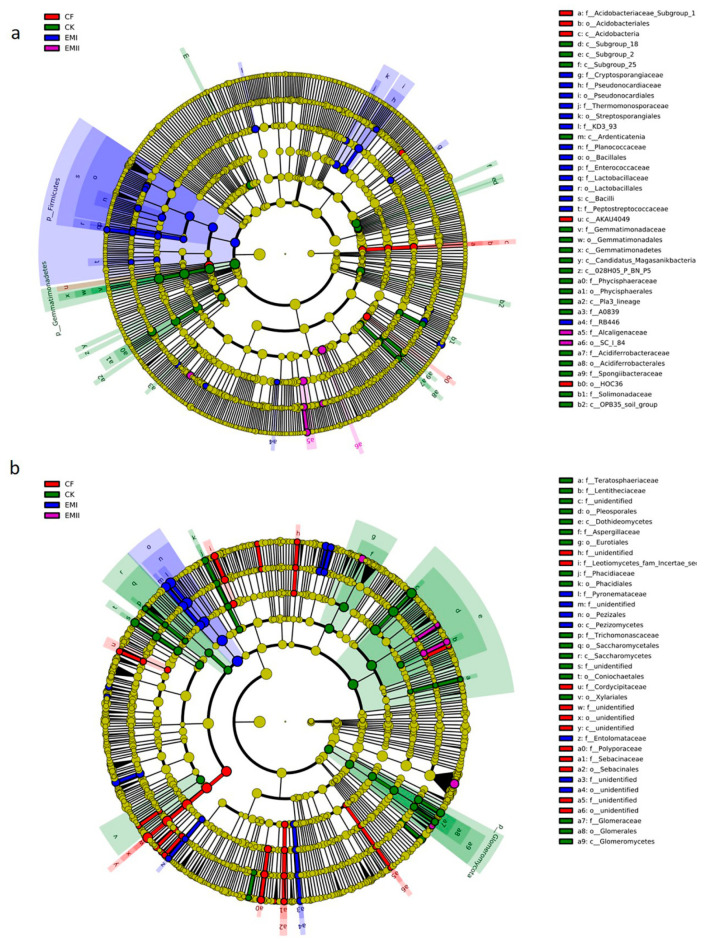
LEfSe analysis of soil bacterial (**a**) and fungal (**b**) biomarkers under different fertilization treatments. Cladogram representing the taxonomic hierarchical structure of the phylotype biomarkers identified among fertilization treatments. Five rings of the cladogram stand for kingdom(innermost), phylum, class, order, family, genus, and species (outermost), respectively. Each small circle represents one biomarker. Each colour means that phylotypes were over represented in corresponding treatment except for yellow. Yellow phylotypes were not significantly different among treatments.

The LEfSe analysis identified high-dimensional biomarker taxa with significantly different abundances in all treatments. Sixty-four biomarkers of bacterial communities were identified (Figure 5a and Figure S1a). Twenty-eight biomarkers in EMI treatment were higher than for the other treatments, mainly including Firmicutes phylum, Bacilli class, and some beneficial genera (*Steroidobacter*, *Kaistia* within Rhizobiaceae, *Sinibacillus*, *Lysinibacillus*, *Lactobacillus*, *Oceanobacillus*, etc.). SC_I_84 order within Betaproteobacteria, and four genera were enriched in EMII treatment. Acidobacteria and its taxa (Acidobacteriales, Subgroup_1 family and Subgroup_7 order) were enriched in CF treatment. Some taxa within Gemmatimonadetes (from phylum to family), and certain classes of Acidobacteria (including Subgroup_25, Subgroup_2 and Subgroup_28) were significantly enriched in CK-treated soil.

A total of 105 biomarkers of fungi were identified (Figure 5b and Figure S1b). *Pezizomycetes*, *Pezizales*, *Cercophora* genus and three species (*Pezizales* sp., *Pyronemataceae* sp. and *Cercophora_samala*) within Ascomycota were significantly enriched in the EMI treatment. Two genera (*Sclerostagonospora* and *Setoseptoria*) and six species (such as *Mortierella_indohii* and *Penicillium_laeve*) were significantly enriched in EMII treatment. Aspergillaceae was identified as the most abundant biomarkers present in CK treatment. Some taxa within Ascomycota and Basidiomycota were significantly enriched in CF treatment.

### 3.3. Relationships between Microbial Community Composition and Soil Properties

As shown in Figure S2, NO_3_^−^-N and AP are the major factor affecting the bacterial and fungal community diversity, respectively. Most of the soil properties (such as TP, TN, NO_3_^−^-N, AP, AK, SOM and EC) increased soil bacterial and fungal richness and decreased their diversity, while pH, WC, and NH_4_^+^-N were inconsistent. Soil pH increased soil bacterial richness and decreased diversity, while the opposite effect was found for fungi. NH_4_^+^-N and WC increased soil bacterial richness and diversity. WC decreased soil fungal richness and diversity, while NH_4_^+^-N increased fungal richness and decreased their diversity. 

**Figure 6:**
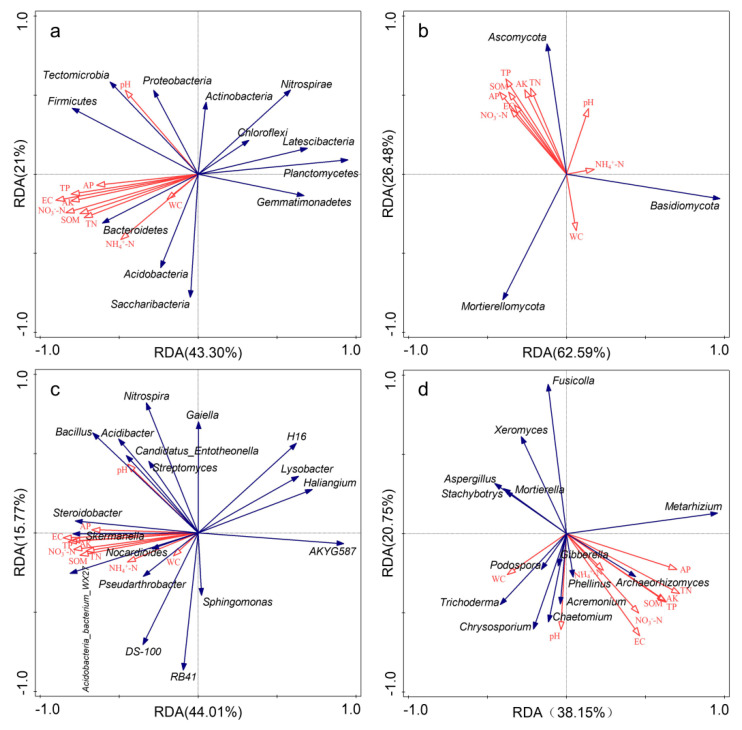
Redundancy analysis (RDA) between soil properties and microbial community composition (relative abundance > 1%) under different fertilization treatments. Red arrows represent soil properties; blue arrows represent different species. (**a**): bacterial phyla; (**b**): fungal phyla; (**c**): bacterial genera; (**d**): fungal genera.

RDA results showed that the first two axes explained, respectively, 64.30% and 59.78% of the total variation of soil bacterial communities at the phylum and genus level (Figure 6a,c). RDA and Mantel test results showed that EC was the most important factor affecting the soil bacterial community composition, and EC, SOM, TP, NO_3_^−^-N and AK were also significantly correlated with soil bacterial community composition (Table 2). As shown in Figure 6b,d, the first two axes explained 89.07% and 58.90% of total variation of soil fungal communities at the phylum and genus level. AK appeared to be the main factor influencing soil fungal community composition at the phylum level, TN (*p* = 0.024), EC (*p* = 0.008) and pH (*p* = 0.05) appeared to be the main factors significantly influencing soil fungal community at the genus level. Soil fungal community composition was significantly correlated with pH, EC, SOM, TP, NO_3_^−^-N and AK (Table 2). Spearman’s correlation analysis showed that bacteria were more sensitive to changes in soil properties than fungi (Figures S3 and S4).

### 3.4. Network Analysis of Microbial Communities

**Figure 7:**
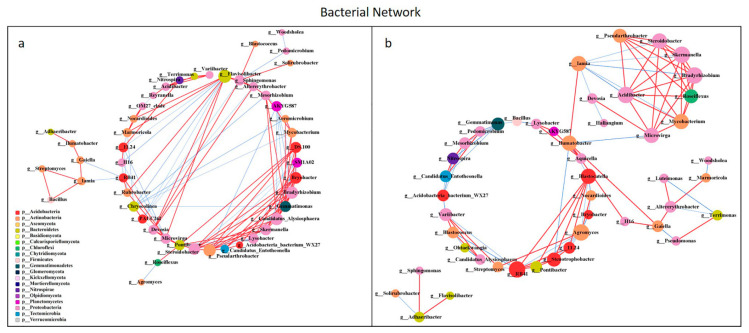
Network analysis showing the co-occurrence of bacterial communities under non-bio-compost (**a**) and bio-compost (**b**) treatments. The co-occurring networks are colored by microbial taxonomic information at the phylum level. The red lines represent significantly positive (*r* > 0.6) interrelationships, blue lines represent negative (*r* < −0.6) interrelationships. The size of the node represents the degree, and the width of the lines represents the strength of the correlation. Non-bio-compost treatments include CK and CF, and bio-compost treatments include EMI and EMII.

**Figure 8:**
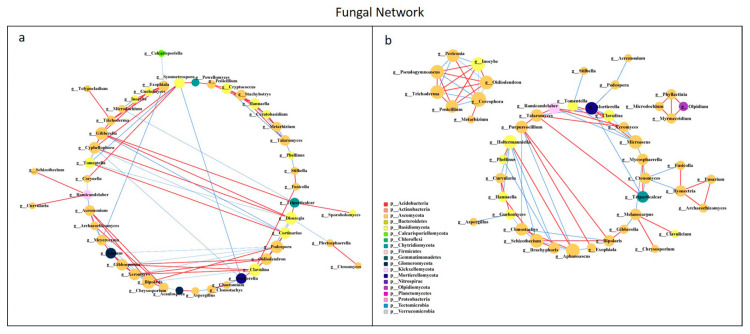
Network analysis showing the co-occurrence of fungal communities under non-bio-compost (**a**) and bio-compost (**b**) treatments. The co-occurring networks are colored by microbial taxonomic information at the phylum level. The red lines represent significantly positive (*r* > 0.6) interrelationships, blue lines represent negative (*r* < −0.6) interrelationships. The size of the node represents the degree, and the width of the lines represents the strength of the correlation. Non-bio-compost treatments include CK and CF, and bio-compost treatments include EMI and EMII.

All network parameters except the average betweenness centrality of bacteria were larger than that of fungi, and there were more positive links than negative links (Figure S5, Table S5). Bio-compost affected the bacterial and fungal co-occurrence patterns (Figure 7 and Figure 8). Positive links of the bacterial network were 37.20–55.10% higher than fungi network. Under bio-compost treatments, there were a greater number of positive links, average degree and average betweenness centrality among microbial communities in bacteria than that in fungi. Compared with non-bio-compost treatments, bio-compost treatments decreased the positive links and average degree and increased the average cluster coefficient, betweenness centrality and closeness centrality in bacterial and fungal networks. Bio-compost changed key nodes in the network structure.

### 3.5. Functional Prediction of Soil Bacterial and Fungal Communities

#### 3.5.1. Functional Prediction of Soil Bacterial Communities

**Figure 9:**
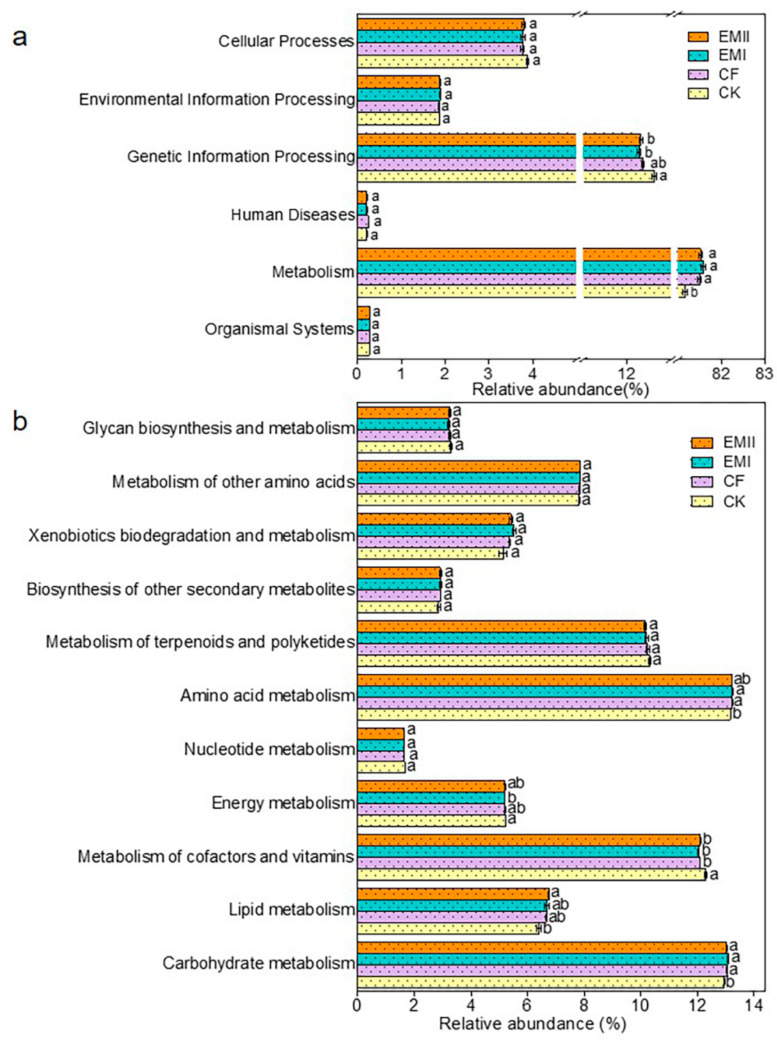
The relative abundance of the predicted KEGG Orthologs functional profiles (KEGG level 1) (**a**) and metabolic functions (**b**) of bacterial communities under different fertilization treatments. Different letters indicate significant differences under different fertilization treatments (*p* < 0.05).

The PICRUSt prediction results showed that Metabolism (81.16–81.58%) was the primary pathway, following the trend EMI > EMII > CF > CK (Figure 9a). Among the secondary functional layers of the Metabolism, the relative abundance of Carbohydrate metabolism in EMI and EMII treatments, Lipid metabolism in EMII treatment, and Amino acid metabolism in EMI treatment was higher than that of CK (Figure 9b). For the tertiary functional layer, the proportion of Nitrogen metabolism in EMI and EMII treatments was higher than that of CK, while D-Glutamine and D-glutamate metabolism, Carbon fixation in photosynthetic organisms, and one-carbon pool by folate were the opposite; and the proportion of Sphingolipid metabolism under EMII treatment was higher than that of CF (Figure S6). The relative abundance of key genes related to carbon (C), nitrogen (N), and phosphorus (P) cycles was predicted (Table S6). In addition, the metabolic function of bacteria was closely related to the dominant microorganisms of bacteria (Tables S7–S12). The above results indicated that the application of the bio-compost altered the soil bacterial-community metabolic function, especially the metabolic capacities related to microbes with respect to soil C, N and P cycles.

#### 3.5.2. Functional Prediction of Soil Fungal Communities

**Figure 10:**
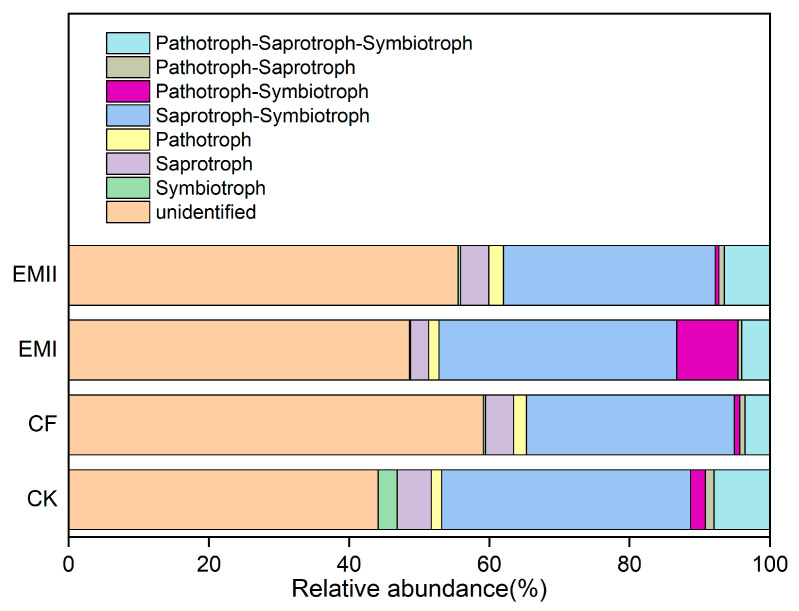
Relative abundance of fungal functional groups (guilds) under different fertilization treatments.

FunGuild was used to predict the nutritional and functional groups of fungal communities. Six trophic mode groups were classified, with Saprotroph–Symbiotroph (29.65–35.47%) being the major components (Figure 10). The ratios of soil Dung Saprotroph-Ectomycorrhizal-Soil Saprotroph-Wood Saprotroph and Dung Saprotroph under the bio-compost treatments were significantly higher than those of CK and CF treatments (Figure S7). However, the ratio of Arbuscular Mycorrhizal under the EMI treatment was significantly higher than those of CK and CF treatments (Figure S7).

## 4. Discussion

### 4.1. High Dosage of Bio-Compost (EMI) Had a Greater Impact on Soil Microbial Alpha-Diversity 

The richness and diversity of the microbial community play a critical role in the functions of soil, and they can be affected by fertilization. The Chao1 and Shannon indices describe the richness and diversity of microbial community, respectively, with the larger values indicating greater richness and diversity [26]. Our results have demonstrated that a high dosage of bio-compost had a greater impact on bacterial and fungal richness than CF and EMII treatments, which implied that bacterial and fungal richness were affected by the dosage of compost and the type of amendments [27,28]. The bacterial-richness index increased with increasing dosages of bio-compost, which could be attributed to the added nutrients with the compost application. Interestingly, fungal richness showed no dose-dependent effect by bio-compost. It was generally believed that microbial diversity increased with manure application [28,29], while our results showed that a high dosage of bio-compost increased fungal richness, while a conventional dosage of bio-compost reduced fungal richness. There were two reasons for this, one was that bio-compost stimulated the proliferation of certain specific microorganisms and inhibited the growth of other microorganisms, and the other was that most soil nutrients were negatively correlated with the fungal chao1 index. Thus, high dosages of bio-compost had a greater impact on soil microbial alpha-diversity.

It has been confirmed that soil physical and chemical factors could drive the changes of soil alpha-diversity [30]. Our results showed that the main factors affecting soil bacterial and fungal diversity were NO_3_^−^-N and AP, which is consistent with previous studies [31]. Most of the soil properties (such as TP, TN, NO_3_^−^-N, AP, AK, SOM and EC) increased soil bacterial and fungal richness, which is also consistent with previous studies [32,33], indicating that the soil microbial-community diversity changes depended on soil nutrient supply. However, the response of the richness and diversity of soil bacteria and fungi to WC, NH_4_^+^-N and pH was different from that to other nutrient indicators. Soil bacterial richness and diversity increased with the increase in WC, and it was proposed that the main reason for this was that bacterial growth can promote a more positive response to the increase in soil moisture by bio-compost [33]. Soil bacterial richness and diversity increased with the increase in NH_4_^+^-N, which may be because the increase in NH_4_^+^-N caused by bio-compost provided a source of nitrogen for microorganism [34]. In addition, the fungi had a wide pH range (5–9) [32], which was conducive to the optimal growth of fungi, explaining that pH may increase fungal diversity. Therefore, this further indicated that soil microorganisms made great contributions to the process of soil nutrient turnover, which in turn affected the soil microbial community.

### 4.2. Significant Effect of Bio-Compost on Soil Microbial Community Composition

Soil microorganisms play a key role in N cycle and organic matter dynamics. Changes in the structure and composition of the soil microbial community will lead to changes in soil quality [35]. It is well known that fertilization can significantly change the soil microbial-community structure and composition [13,33]. Our results showed that the bio-compost application altered the community structure of soil bacteria and fungi, because the specific functional microorganisms in the bio-compost entered the soil and interacted with the indigenous microorganisms of the soil, further altering soil microbial community structure [36].

The RDA and Mantel test results showed that the key soil physicochemical factors (such as EC, SOM, TP, NO_3_^−^-N and AK) affected soil bacterial and fungal community composition at the same time, which is partly consistent with previous studies [13]. Previous researches reported that soil pH had a greater impact on bacterial communities than fungal communities [32,37], but our results showed that soil pH had a greater impact on fungal communities. This may be because the fungi had a larger pH range than bacteria [32], which was conducive to the optimal growth of fungi.

### 4.3. Bio-Compost Changed the Dominant Community Representatives and Biomarkers of Bacteria and Fungi

The bacterial and fungal-dominant community representatives played a key role in soil microbial metabolism, nutrient transformation and crop quality. The shifts in soil microbial communities induced by bio-compost were reflected in changes in dominant community representatives at phylum and genus levels of soil bacteria and fungi, especially the enrichment of beneficial microorganisms in the soil and the reduction of harmful microorganisms.

For bacteria, bio-composts increased the relative abundance of Proteobacteria and some of its genera, which is consistent with Longa et al. [38]. The presence of Proteobacteria is characteristic of nutrient-rich and high-carbon substrates [39], and bio-compost significantly increased the content of soil organic matter and provided a carbon source for Proteobacteria. Bio-composts increased the functional bacteria (such as *Skermanella* and SC_I_84 order within Proteobacteria) participating in the soil N cycle [40]. The significant correlation between Proteobacteria and most metabolic pathways related to N cycle implied that Proteobacteria promoted soil nitrogen transformation. The conventional dosage of bio-compost increased the relative abundance of *Sphingomonas*, which was related to the high content of Sphingolipid metabolism in the conventional dosage of bio-compost. *Sphingomonas* was a functional microbe with strong metabolic ability and can degrade organic pollutants in the soil. Bio-composts increased the relative abundance of Actinobacteria. The reason may be that Actinobacteria participated in the degradation of organic matter, while bio-compost increased organic matter and provided carbon source for Actinobacteria. In addition, bio-composts increased the relative abundance of *Nocardioides*, *Pseudarthrobacter* and *Streptomyces* within Actinobacteria, which belong to beneficial bacteria and can secrete actinomycin and antagonize the growth of soil pathogens [40,41]. 

Bio-composts increased the relative abundance of Bacteroidetes, which can synthesize glycosyl hydrolases to break down cellulose and hemicellulose, help in the soil N cycle and nutrient turnover and be positively related to soil nutrients [42]. Firmicutes was enriched in EMI treatment, and its positive correlation with soil nutrients explained this. Firmicutes was a symbiotic bacteria, which contributed to the C cycle, degraded plant-derived polysaccharides, and had a significant positive correlation with most carbon metabolic pathways [43]. Additionally, the LEfSe analysis also showed a Bacilli order and that other branches (*Oceanobacillus*, *Lysinibacillus*, *Lactobacillaceae*, etc.) were enriched in EMI treatment. The main reason was that the EM agent added to bio-compost was rich in Bacillus (Bacillus licheniformis, Bacillus subtilis, Bacillus glaci, Bacillus thuringiensis, etc.) which can inhibit plant pathogens [12]. 

Soil fungi plays an important and complex role in maintaining the normal operation of biological communities, such as establishing symbiotic or pathogenic relationships with plants and animals, participating in C and N cycles, and promoting the decomposition of organic matter. The most abundant phylum in all treatments was Ascomycota, which contained many beneficial and pathogenic microorganisms. Bio-composts increased the relative abundance of Ascomycota, which may be due to the fact that the bio-compost can provide nutrients for the growth of Ascomycota. Bio-compost increased the relative abundance of beneficial fungi, such as *Chrysosporium*, *Chaetomium*, *Acremonium* and *Trichoderma* within Ascomycota, which were commonly found biocontrol fungi and had inhibitory effects on plant pathogenic microorganisms [12,44]. High dosages of bio-compost increased the relative abundance of *Metarhizium* that can prevent fusarium wilt [45]. Bio-compost decreased the relative abundance of *Stachybotrys*, which is a plant pathogenic fungus and indoor pollution fungus that can easily cause human diseases [46]. High dosage of the bio-compost decreased the relative abundance of *Aspergillus* that can cause ear rot in plants [47]. The LEfSe analysis showed that *Pezizales* within Ascomycota under EMI treatment was significantly higher than other treatments because *Pezizales* preferred habitats with available organic compounds, and are stimulated by compost correction [48]. Bio-compost reduced the relative abundance of Mortierellomycota and *Mortierella*, which was also explained by their negative correlation with soil nutrients. Bio-composts led to a decrease in the relative abundance of Basidiomycota, which is consistent with Sun et al. [49]. It has been reported that Basidiomycetes can rapidly metabolize organic matter in the top soil [50]. 

### 4.4. Bio-Compost Altered Bacterial and Fungal Co-Occurrence Patterns

The application of bio-compost had a significant impact on both the individual microbial groups and the overall microbial community patterns. The bacterial network was more complex than the fungal network, which was in accordance with Li et al. [28]. The average cluster coefficient and closeness centrality in the bacterial and fungal network of bio-compost treatments were higher than that of the non-bio-compost treatments. This implied that the application of bio-composts might not only remit the competition within bacteria, but also reduce competition within fungi. The higher average betweenness centrality of the bacterial and fungal network under bio-compost treatments than non-bio-compost treatments suggested that bio-compost may increase the bacterial and fungal niches. However, bacteria occupied a larger core niche than fungi. The fact that the average betweenness centrality of the bacterial network under bio-compost treatment is higher than that of the fungal network confirmed this. The average closeness centrality of bacterial network under all treatments was greater than that of fungal network, indicating that information transmission between bacteria was greater than between fungi. This implies that bacteria can quickly respond to environmental changes, and Spearman’s correlation analysis also explained this (Figures S3 and S4). Positive links dominated both the bacterial and fungal networks, implying that mutual cooperation rather than competitive exclusion played a more important role in microbial assembly. Mutual cooperation occurred more frequently in bacterial communities than in the fungal network because positive links in the bacterial network were much higher. The application of bio-compost provided a higher supply of nutrients for microorganisms, which reduced competition for limited resources and promoted cooperation between species. Bio-compost changed key nodes in the network structure, because the functional microorganisms of bio-compost entered the soil and interacted with the indigenous microorganisms of the soil.

### 4.5. The Application of Bio-Compost Changed the Overall Function of Soil Bacterial and Fungal Communities

The application of bio-compost changed the overall function of soil bacterial and fungal communities. The PICRUSt results showed that Metabolism was the most important of the six categories of biological metabolic pathways, which was a similar result to Pii et al. [51]. In addition, the application of bio-compost increased the ratio of Metabolism among the six categories of biological metabolic pathways. The higher ratios of Carbohydrate metabolism, lipid metabolism, Xenobiotics biodegradation and metabolism, and amino acid metabolism belonging to metabolism in EMI treatment than CK treatment also suggested this. This result may be because fertilization increased soil nutrients, especially organic carbon, provided abundant metabolic substrates for bacteria, and further changed the metabolic functions of bacterial communities. The proportion of certain metabolic pathways related to C, N and P cycles under bio-compost treatments was larger than that of CK and CF treatments, indicating that the bio-compost shifted the metabolic function of microorganisms. The key genes related to C cycle were mainly involved in C degradation, C fixation, and Methane cycling. The key genes related to N cycle were mainly involved in fixation, ammonification, nitrification, denitrification, dissimilatory N reduction, and assimilatory N reduction. Additionally, the key genes related to P cycle were mainly involved in P oxidation, Phytic acid hydrolysis, polyphosphate degradation, and polyphosphate synthesis. Due to limitations of PICRUSt analysis, the functions of related bacteria were only preliminarily predicted in our study. In future research, metagenomics and other methods will be used for further verification. 

The FUNGuild results showed that six trophic mode groups could be classified, which is consistent with Chen et al. [52]. The Saprotroph–Symbiotroph was the major trophic mode, the fungi for which played great roles in decomposing organic matter and nutrient cycle, and improving crop quality. At the same time, bio-compost significantly increased the ratios of soil Dung Saprotroph-Ectomycorrhizal-Soil Saprotroph-Wood Saprotroph and Dung Saprotroph that belong to Saprotroph–Symbiotroph and Saprotroph, respectively. This result suggested bio-compost altered fungal trophic modes.

Overall, the application of the bio-compost altered the soil bacterial and fungal community function, their metabolic function and trophic modes.

## 5. Conclusions

A 25-year application of high dosages of bio-compost significantly increased bacterial and fungal richness by 7.11% and 5.71%, respectively, and altered the composition of bacterial and fungal communities. The enrichment of beneficial microorganisms (such as *Sphingomonas*, *Acidibacter*, *Streptomyces*, *Oceanobacillus*, etc.) and the reduction of harmful microorganisms (such as *Stachybotrys* and *Aspergillus*) was observed in the bio-compost-treated soil. The complexity of the bacterial network was higher than fungi, and bacteria were more sensitive to changes in environmental factors than fungi. Positive links of the bacterial network were 37.20–55.10% higher than the fungi network. Bio-composts remit the competition of individual strains within bacteria and fungi. The functional prediction of soil bacteria and fungi suggested that bio-composts altered the soil bacterial-community metabolic function (especially metabolic capacities related to microbes with respect to soil C, N and P cycles) and the soil fungal-community function (mainly trophic modes). In short, our findings suggest that suitable bio-compost addition is helpful for soil health and crop growth.

## Figures and Tables

**Table 1:** The composition and content of microorganisms in effective microorganism (EM) agent solution.

	Contents (10^8^ cfu/mL)
*Bacillus megaterium*	0.157
*Saccharomyces cerevisiae*	0.0043
*Lactobacillus plantarum*	3.730

**Table 2:** Mantel tests of the soil bacterial and fungal communities with soil properties under different treatments.

	Bacteria		Fungi	
	*r*	*p*	*r*	*p*
WC	0.0217	0.411	−0.1025	0.736
pH	0.1223	0.245	0.3483	0.038
EC	0.5491	0.003	0.5596	0.001
SOM	0.4794	0.009	0.4175	0.007
TP	0.5281	0.001	0.4582	0.004
TN	0.3789	0.069	0.3197	0.050
NO_3_^−^-N	0.4927	0.022	0.3800	0.009
NH_4_^+^-N	0.1162	0.196	−0.0131	0.505
AP	0.3300	0.054	0.2603	0.059
AK	0.4914	0.01	0.4116	0.006

WCWC: soil water content; EC: electrical conductivity; SOM: soil organic matter; TP: total phosphorus; TN: total nitrogen; NO_3_^−^-N: nitrate nitrogen; NH_4_^+^-N: ammonium nitrogen; AP: available phosphorus; AK: available potassium.

## Data Availability

The datasets of bacterial and fungal sequences generated for this study can be found in the Sequence Read Archive (SRA) data of National Center for Biotechnology Information under accession numbers SRP336958 and SRP336961, respectively.

## Supplementary Materials

The following supporting information can be downloaded at: https://www.mdpi.com/article/10.3390/microorganisms10020462/s1, Table S1: The soil background value before the experiment, Table S2: Information of selected forward (F) and reverse (R) primers targeting different type of genes for qPCR, Table S3: The relative abundance of soil bacterial communities at different phylum levels under different fertilization treatments, Table S4: The relative abundance of soil fungal communities at different phylum levels under different fertilization treatments, Table S5: Microbial network parameters, Table S6: KEGG Orthologies related to C, N and P cycles, Table S7: The Pearson’s correlation analysis between soil bacterial phyla and key metabolic pathways related to the C cycle, Table S8: The Pearson’s correlation analysis between soil bacterial phyla and key metabolic pathways related to the N cycle, Table S9: The Pearson’s correlation analysis between soil bacterial phyla and key metabolic pathways related to the P cycle, Table S10: The Pearson’s correlation analysis between soil bacterial genera and key metabolic pathways related to the C cycle, Table S11: The Pearson’s correlation analysis between soil bacterial genera and key metabolic pathways related to the N cycle, Table S12: The Pearson’s correlation analysis between soil bacterial genera and key metabolic pathways related to the P cycle; Figure S1: The linear discriminant analysis (LDA) effect size (LEfSe) of soil bacterial (a) and fungal (b) biomarkers under different fertilization treatments. Identified phylotype biomarkers were ranked by effect size (> 3), and the α value was < 0.05, Figure S2: Redundancy analysis (RDA) between soil bacterial (a) and fungal (b) alpha-diversity and soil properties under different fertilization treatments. Red arrows represent soil properties; blue arrows represent alpha-diversity, Figure S3: The heatmap of the correlation between soil properties and the relative abundance of bacterial (a) and fungal (b) phylum under different fertilization treatments (relative abundance > 1%). This heatmap was created according to the result of Spearman’s correlation analysis. Positive relationships are represented in red, while negative relationships are represented in blue. The significant correlations are presented as asterisks (*, p < 0.05; **, *p* < 0.01), Figure S4: The heatmap of the correlation between soil properties and the relative abundance of bacterial (a) and fungal (b) genera under different fertilization treatments (relative abundance > 1%). This heatmap was created according to the result of Spearman’s correlation analysis. Positive relationships are represented in red, while negative relationships are represented in blue. The significant correlations are presented as asterisks (*, *p* < 0.05; **, *p* < 0.01), Figure S5: Network analysis showing overall bacterial (a) and fungal (b) co-occurrence. The co-occurring networks are colored by microbial taxonomic information at the phylum level. The red lines represent significantly positive (*r* > 0.6) interrelationships, while blue lines represent negative (*r* < −0.6) interrelationships. The size of the node represents the abundance, and the width of the lines represents the strength of the correlation, Figure S6: Extended error bar plots showing statistically significant differences in the bacterial functional groups between the fertilization treatments and the control treatment. Error bars indicate within-group standard deviations. Presented categories passed a corrected *p* value of < 0.05 in Welch’s t test, Figure S7: Extended error bar plots showing statistically significant differences in the fungal functional groups (guilds) between the fertilization treatments and the control treatment. Error bars indicate within-group standard deviations. Presented categories passed a corrected *p* value of < 0.05 in Welch’s t test.
