# Supplementary material for: Long-Term Application of Bio-Compost Increased Soil Microbial Community Diversity and Altered Its Composition and Network
Source: Microorganisms. 2022 Feb 17;10(2):462. doi: 10.3390/microorganisms10020462 (PMC8878586; doi:10.3390/microorganisms10020462)
Supplement: Supplementary file 1 [file microorganisms-10-00462-s001.zip › microorganisms-1588024-supplementary.pdf]

Supplementary Information

Long-Term Application of Bio-Compost Increased Soil Microbial Community Diversity and Altered its Composition and Network

Xiayan Liu [1], Yu Shi [2], Lingyu Kong [1], Lihong Tong [3], Haoxuan Cao [1], Hu Zhou [1,*] and Yizhong Lv [1,*]

[1] Department of Soil and Water Sciences, College of Land Science and Technology,
China Agricultural University, Beijing 100193, China; liuxiayan@cau.edu.cn (X.L.);
13427569158@163.com (L.K.); caohaoxuan21@163.com (H.C.)
[2] State Key Laboratory of Crop Stress Adaptation and Improvement, School of Life Sciences,
Henan University, Kaifeng 475004, China; yshi@henu.edu.cn
[3] State Key Laboratory of Hydroscience and Engineering, Department of Hydraulic Engineering,
Tsinghua University, Beijing 100084, China; tonglihong@mail.tsinghua.edu.cn
* Correspondence: zhouhu@cau.edu.cn (H.Z.); lyz@cau.edu.cn (Y.L.);
Tel.: +86-010-62731890 (H.Z.); +86-010-62731431 (Y.L.)

Table S1. The soil background value before the experiment.

Soil properties	value
pH	7.974
soil organic matter (g kg^{-1})	13
total phosphorus (g kg^{-1})	1.55
total nitrogen (g kg^{-1})	0.84
alkali-hydro nitrogen (mg kg^{-1})	55.62
available phosphorus (mg kg^{-1})	4.71
available potassium (mg kg^{-1})	102.30
Soil bulk density (g cm^{-3})	1.510
caption exchange capacity (cmol kg^{-1})	9.475

Table S2. Information of selected forward (F) and reverse (R) primers targeting different type of genes for qPCR

Target gene	Primer	Primer sequence
Bacterial 16S rRNA	338F	ACTCCTACGGGAGGCAGCAG
	806R	GGACTACHVGGGTWTCTAAT
Fungal ITS	ITS1F	CTTGGTCATTTAGAGGAAGTAA
	ITS2R	TGCGTTCTTCATCGATGC

Table S3. The relative abundance of soil bacterial communities at different phylum levels under different fertilization treatments

	Acidobacteria	Proteobacteria	Actinobacteria	Chloroflexi	Gemmatimonadetes	Bacteroidetes	Planctomycetes	Nitrospirae	Firmicutes	Tectomicrobia	Latescibacteria	Saccharibacteria
CK	29.66a	25.50a	12.22a	9.51a	7.52a	2.77a	3.44a	2.40a	0.99b	0.89a	1.51a	1.02a
CF	34.01a	25.20a	13.34a	8.64a	6.75a	2.57a	1.42b	1.74a	1.13b	1.47a	0.86ab	0.89a
EMI	33.97a	26.21a	13.00a	9.29a	4.17c	3.28a	1.42b	1.59a	1.97a	1.54a	0.82ab	0.91a
EMII	32.62a	27.68a	12.78a	8.70a	5.47b	3.60a	1.24b	1.69a	1.75ab	1.51a	0.59b	0.72a

Values are means for triplicate replicates. Different lowercase letters indicate significant differences at $p < 0.05$ based on the analysis of variance. CK: control treatment without any fertilizer; CF: chemical fertilizer; EMI: high level of biological compost; EMII: conventional level of biological compost.

Table S4. The relative abundance of soil fungal communities at different phylum levels under different fertilization treatments

	Ascomycota	Mortierellomycota	Basidiomycota
CK	42.34a	33.74a	4.00a
CF	44.51a	24.98a	6.25a
EMI	57.15a	21.28a	1.33a
EMII	48.72a	24.02a	1.80a

Values are means for triplicate replicates. Different lowercase letters indicate significant differences at $p < 0.05$ based on the analysis of variance. CK: control treatment without any fertilizer; CF: chemical fertilizer; EMI: high level of biological compost; EMII: conventional level of biological compost.

Table S5. Microbial network parameters

	Network parameters	Overall treatments	bio-compost	Non-bio-compost
Bacteria	Total nodes	49	47	48
	Positive links/Total links	82/123	59/97	76/117
	Negative links/Total links	41/123	38/97	41/117
	Average degree	5.02	4.13	4.88
	Average clustering coefficient	0.34	0.49	0.46
	Average betweenness centrality	0.04	0.09	0.06
	Average closeness centrality	0.41	0.29	0.28
Fungi	Total nodes	44	46	48
	Positive links/Total links	46/83	43/83	49/92
	Negative links/Total links	37/83	40/83	43/92
	Average degree	3.77	3.61	3.83
	Average clustering coefficient	0.31	0.49	0.32
	Average betweenness centrality	0.07	0.08	0.07

	Average closeness centrality	0.26	0.42	0.26

Table S6. KEGG Orthologies related to C, N and P cycles

Gene/Enzyme	KEGG Orthology Numbers											
Related to the C cycle	K01692	K01784	K00626	K01834	K01652	K01703	K01491	K01704	K00128	K01754	K01738	K00820
	K04042	K01649	K00845	K00382	K01961	K00615	K00074	K01091	K01647	K01895	K00249	K01625
	K01092	K01840	K01179	K00135	K00123	K00140	K00100	K00012	K00104	K00812	K00688	K00134
	K01759	K01644	K00240	K01782	K00948	K00239	K00640	K01681	K01187	K00161	K00850	K01783
	K01207	K00600	K02437	K00790	K01689	K00036	K01803	K00830	K01653	K00927	K00075	K00162
	K00605	K00873	K00658	K00241	K00024	K00031	K01809	K00121	K00616	K05349	K01711	K01903
	K01902	K12410	K00001	K00244	K00627	K00847	K01963	K03431	K01791	K00975	K00963	K01810
	K01966	K01808	K00248	K00033	K13953	K00705	K01006	K01623	K01057	K07407	K01512	K00175
	K00174	K01962	K03821	K01595	K05350	K01848	K00124	K00854	K00925	K00008	K00965	K00966
	K07516	K00703	K00851	K01209	K00831	K01190	K00117	K01489	K02446	K01676	K01858	K01624

	K01619	K01628	K00261	K00163	K12308	K01849	K01181	K01069	K01835	K00283	K00282	K01183
	K01785	K00034	K00297	K01938	K00114	K00700	K00164	K02160	K00971	K01029	K00754	K07246
	K01188	K00852	K00978	K00823	K01007	K07106	K01679	K13810	K01443	K02377	K00849	K14138
	K03781	K00176	K01433	K00030	K01638	K01198	K12454	K07250	K00029	K00874	K01816	K01709
	K01654	K00625	K11261	K01053	K03737	K01958	K01640	K05343	K01079	K01752	K00010	K01178
	K00131	K00697	K12452	K01847	K00050	K11473	K00027	K09796	K01087	K00015	K00101	K01807
	K00242	K00042	K02564	K00065	K05606	K01610	K04072	K11263	K00929	K01596	K03335	K01805
	K01965	K01028	K00190	K00023	K01812	K07243	K01637	K03388	K07406	K03738	K03738	K00019
	K00156	K01715	K00281	K00882	K01677	K01678	K05973	K01907	K11472	K03841	K00886	K01039
	K01040	K01685	K04073	K00865	K00177	K01112	K00064	K12972	K02793	K01195	K07404	K01093
	K01684	K00170	K00895	K01908	K00172	K01960	K03336	K00127				
Related to the	K00266	K01915	K01953	K01673	K00265	K00605	K00459	K01745	K00261	K00363	K00034	K01760
N cycle	K01916	K00262	K01424	K01744	K02575	K00285	K00284 K00362	K00260	K14155	K00372	K00366	K02591
	K02586	K02588	K01425	K01758	K02585	K01428						
Related to the	K00655	K01524	K01507	K01507	K01090	K01091	K03574	K01092	K01104	K02036	K06153	K02037

P cycle	K02038	K07636	K07042	K07024	K01520	K06217	K01113	K03820	K03306	K07053	K03270	K01512
	K00937	K08296	K00324	K06881	K07658	K06949	K02446	K07315	K04750	K01095	K03273	K05946
	K06896	K01078	K02221	K07657	K01079	K00325	K03426	K01087	K06193	K01077	K01077	K07313
	K03841	K01112	K01093	K04486	K01525							

Table S7. The Pearson's correlation analysis between soil bacterial phyla and key metabolic pathways related to the C cycle

	Acidobacteria	Proteobacteria	Actinobacteria	Chloroflexi	Gemmatimonadetes	Bacteroidetes	Planctomycetes	Nitrospirae	Firmicutes	Tectomicrobia	Latescibacteria	Saccharibacteria
Glycine, serine and threonine metabolism	.734**	-.762**	-0.327	0.321	-0.13	-0.444	-0.077	0.033	-0.449	-0.282	0.089	-0.239
Cysteine and methionine metabolism	.827**	-.798**	-.876**	0.141	0.038	0.088	0.063	-0.281	-0.369	-0.423	0.162	0.101
Valine, leucine and isoleucine biosynthesis	0.092	-0.519	-0.064	.708**	0.308	-.665*	0.569	.748**	-0.537	-0.467	.637*	-0.495
Arginine and proline metabolism	0.191	0.268	0.183	-0.166	-.837**	0.097	-.714**	-0.393	.585*	.722**	-.619*	-0.227
Tryptophan metabolism	-0.188	.645*	0.508	-0.495	-.621*	0.348	-.778**	-0.504	.684*	.719**	-.775**	0.068
Glycolysis / Gluconeogenesis	-.932**	.785**	.734**	-0.025	0.152	0.05	0.039	0.268	0.426	0.503	0.036	-0.03
Citrate cycle (TCA cycle)	0.01	-0.353	-0.016	0.15	.667*	-0.226	0.43	0.378	-.618*	-.801**	0.378	0.07
Pentose phosphate pathway	.712**	-.881**	-0.44	0.575	-0.171	-0.292	0.123	-0.039	-0.341	-0.38	0.241	-0.049
Pentose and glucuronate interconversions	0.421	0.141	-0.153	-0.44	-.795**	.599*	-.816**	-.905**	.604*	0.5	-.802**	0.312
Fructose and mannose metabolism	.973**	-.841**	-0.563	0.132	-0.311	-0.152	-0.191	-0.382	-0.341	-0.347	-0.147	0.082
Galactose metabolism	.834**	-.714**	-0.514	0.007	-0.229	0.102	-0.284	-.659*	-0.178	-0.224	-0.244	0.43
Ascorbate and aldarate metabolism	-0.527	.846**	0.494	-0.322	-0.475	0.299	-0.477	-0.162	.771**	.864**	-0.433	-0.148
Starch and sucrose metabolism	-0.23	0.392	0.169	-0.254	-0.25	0.393	-0.394	-0.53	0.503	.594*	-0.351	0.46
Amino sugar and nucleotide sugar metabolism	.933**	-.910**	-.685*	0.175	-0.1	-0.119	-0.039	-0.368	-0.432	-0.425	0.019	0.193
Inositol phosphate metabolism	-0.392	.585*	.672*	-0.196	-0.463	0.143	-0.54	-0.337	.602*	.677*	-0.558	0.218
Pyruvate metabolism	-.799**	.844**	0.561	-0.065	-0.12	0.114	-0.119	0.212	.629*	.761**	-0.027	-0.287
Glyoxylate and dicarboxylate metabolism	-0.135	.580*	0.43	-0.271	-.690*	0.119	-.671*	-0.162	.608*	.698*	-.622*	-0.333
Propanoate metabolism	0.499	0.041	0.016	-0.324	-.800**	0.283	-.874**	-.659*	0.48	0.444	-.725**	-0.067
Butanoate metabolism	-0.46	.765**	.715**	-0.327	-0.49	0.157	-.577*	-0.18	.649*	.714**	-0.575	-0.089
C5-Branched dibasic acid metabolism	-0.243	-0.246	0.138	.723**	0.397	-.642*	.647*	.914**	-0.395	-0.346	.724**	-0.571
Methane metabolism	-.784**	.913**	.642*	-0.169	-0.213	0.161	-0.23	0.164	.664*	.747**	-0.195	-0.27
Carbon fixation in photosynthetic organisms	0.051	-0.561	-0.379	0.448	.745**	-0.337	.793**	0.534	-.712**	-.725**	.778**	-0.01

Carbon fixation pathways in prokaryotes	.747**	-.903**	-.632*	0.314	0.229	-0.254	0.26	0.067	-.630*	-.724**	0.342	-0.058
ABC transporters	-0.528	.722**	.613*	-0.041	-0.49	0.023	-0.383	0.055	.677*	.827**	-0.328	-0.311
One carbon pool by folate	-0.441	-0.05	-0.053	0.228	.832**	-0.133	.807**	.625*	-0.453	-.582*	.754**	0
Cyanoamino acid metabolism	0.325	0.176	0.252	-0.328	-.776**	0.16	-.848**	-0.556	0.407	0.455	-.880**	0.032

A value greater than zero represents a positive correlation, a value less than zero represents a negative correlation. The significant correlations are presented as asterisks (*, $p < 0.05$;**, $p < 0.01$).

Table S8. The Pearson's correlation analysis between soil bacterial phyla and key metabolic pathways related to the N cycle

	Acidobacteria	Proteobacteria	Actinobacteria	Chloroflexi	Gemmatimonadetes	Bacteroidetes	Planctomycetes	Nitrospirae	Firmicutes	Tectomicrobia	Latescibacteria	Saccharibacteria
Alanine, aspartate and glutamate metabolism	0.191	-.682*	-0.413	0.497	.663*	-0.412	.710**	0.499	-.671*	-.658*	.836**	-0.153
Glycine, serine and threonine metabolism	.734**	-.762**	-0.327	0.321	-0.13	-0.444	-0.077	0.033	-0.449	-0.282	0.089	-0.239
Cysteine and methionine metabolism	.827**	-.798**	-.876**	0.141	0.038	0.088	0.063	-0.281	-0.369	-0.423	0.162	0.101
Arginine and proline metabolism	0.191	0.268	0.183	-0.166	-.837**	0.097	-.714**	-0.393	.585*	.722**	-.619*	-0.227
Histidine metabolism	.895**	-.886**	-.669*	0.272	-0.113	-0.125	0.01	-0.145	-0.404	-0.517	0.15	-0.077
Tyrosine metabolism	0.18	0.407	0.067	-.647*	-.652*	0.529	-.837**	-.819**	0.574	.615*	-.879**	0.296
Phenylalanine metabolism	-0.197	.655*	0.489	-0.469	-.613*	0.257	-.746**	-0.444	.629*	.771**	-.795**	0.041
Tryptophan metabolism	-0.188	.645*	0.508	-0.495	-.621*	0.348	-.778**	-0.504	.684*	.719**	-.775**	0.068
Pentose phosphate pathway	.712**	-.881**	-0.44	0.575	-0.171	-0.292	0.123	-0.039	-0.341	-0.38	0.241	-0.049
C5-Branched dibasic acid metabolism	-0.243	-0.246	0.138	.723**	0.397	-.642*	.647*	.914**	-0.395	-0.346	.724**	-0.571
Nitrogen metabolism	.583*	-0.007	-0.416	-0.359	-.727**	0.537	-.780**	-.772**	0.534	0.496	-.602*	0.007
One carbon pool by folate	-0.441	-0.05	-0.053	0.228	.832**	-0.133	.807**	.625*	-0.453	-.582*	.754**	0
Nicotinate and nicotinamide metabolism	.614*	-.863**	-0.293	0.487	0.15	-0.475	0.26	0.271	-.606*	-.704*	0.366	-0.219
Taurine and hypotaurine metabolism	.851**	-0.358	-0.529	-0.371	-0.521	0.274	-.651*	-.735**	0.047	0.137	-.591*	0.14
Selenocompound metabolism	.982**	-.828**	-.599*	0.04	-0.24	-0.061	-0.225	-0.455	-0.38	-0.403	-0.21	0.196

Cyanoamino acid metabolism	0.325	0.176	0.252	-0.328	-.776**	0.16	-.848**	-0.556	0.407	0.455	-.880**	0.032
D-Glutamine and D-glutamate metabolism	-0.189	-0.385	-0.135	0.393	.866**	-0.392	.820**	.622*	-.680*	-.697*	.823**	0.02
Purine metabolism	-0.035	-0.536	-0.224	0.543	.721**	-0.434	.815**	.646*	-.622*	-.681*	.893**	-0.174
Two-component system	-.612*	.900**	0.171	-0.469	-0.136	0.539	-0.228	-0.083	.689*	.670*	-0.173	-0.122
Chloroalkane and chloroalkene degradation	-0.299	.752**	0.454	-0.445	-.623*	0.374	-.702*	-0.397	.764**	.855**	-.685*	-0.059
Aminobenzoate degradation	-0.412	.718**	.707*	-0.341	-0.506	0.082	-0.57	-0.201	.590*	.729**	-.602*	-0.056
Atrazine degradation	-0.392	.674*	0.54	-0.176	-0.549	0.018	-0.457	-0.089	.616*	.842**	-0.471	-0.21

A value greater than zero represents a positive correlation, a value less than zero represents a negative correlation. The significant correlations are presented as asterisks (*, $p < 0.05$;**, $p < 0.01$).

Table S9. The Pearson's correlation analysis between soil bacterial phyla and key metabolic pathways related to the P cycle

	Acidobacteria	Proteobacteria	Actinobacteria	Chloroflexi	Gemmatimonadetes	Bacteroidetes	Planctomycetes	Nitrospirae	Firmicutes	Tectomicrobia	Latescibacteria	Saccharibacteria
Glycine, serine and threonine metabolism	.734**	-.762**	-0.327	0.321	-0.13	-0.444	-0.077	0.033	-0.449	-0.282	0.089	-0.239
Cysteine and methionine metabolism	.827**	-.798**	-.876**	0.141	0.038	0.088	0.063	-0.281	-0.369	-0.423	0.162	0.101
Histidine metabolism	.895**	-.886**	-.669*	0.272	-0.113	-0.125	0.01	-0.145	-0.404	-0.517	0.15	-0.077
Streptomycin biosynthesis	0.406	-.871**	-0.391	.583*	0.472	-0.531	.605*	0.386	-.744**	-.712**	.670*	-0.048
Glycolysis / Gluconeogenesis	-.932**	.785**	.734**	-0.025	0.152	0.05	0.039	0.268	0.426	0.503	0.036	-0.03
Fructose and mannose metabolism	.973**	-.841**	-0.563	0.132	-0.311	-0.152	-0.191	-0.382	-0.341	-0.347	-0.147	0.082
Galactose metabolism	.834**	-.714**	-0.514	0.007	-0.229	0.102	-0.284	-.659*	-0.178	-0.224	-0.244	0.43
Starch and sucrose metabolism	-0.23	0.392	0.169	-0.254	-0.25	0.393	-0.394	-0.53	0.503	.594*	-0.351	0.46
Amino sugar and nucleotide sugar metabolism	.933**	-.910**	-.685*	0.175	-0.1	-0.119	-0.039	-0.368	-0.432	-0.425	0.019	0.193
Pyruvate metabolism	-.799**	.844**	0.561	-0.065	-0.12	0.114	-0.119	0.212	.629*	.761**	-0.027	-0.287
Glyoxylate and dicarboxylate metabolism	-0.135	.580*	0.43	-0.271	-.690*	0.119	-.671*	-0.162	.608*	.698*	-.622*	-0.333
Oxidative phosphorylation	0.36	-0.548	-0.57	0.2	0.458	0.001	0.426	0.309	-0.534	-.821**	0.489	-0.136
Methane metabolism	-.784**	.913**	.642*	-0.169	-0.213	0.161	-0.23	0.164	.664*	.747**	-0.195	-0.27
Carbon fixation in photosynthetic organisms	0.051	-0.561	-0.379	0.448	.745**	-0.337	.793**	0.534	-.712**	-.725**	.778**	-0.01
RNA degradation	-0.211	0.016	-0.284	0.061	0.445	0.274	0.556	0.383	-0.167	-.633*	0.505	-0.029
Lipopolysaccharide biosynthesis	.684*	-.776**	-.831**	0.236	0.212	-0.085	0.324	0.054	-0.491	-0.549	0.424	-0.142
Glycerolipid metabolism	-0.179	0.4	0.065	-0.242	-0.408	0.54	-0.375	-0.52	.731**	.634*	-0.187	0.175
Glycerophospholipid metabolism	0.435	-0.544	-.580*	0.206	0.258	-0.053	0.268	0.26	-0.328	-0.449	0.575	-0.451
Arachidonic acid metabolism	0.336	0.119	-0.182	-.631*	-0.285	0.533	-.695*	-.876**	0.254	0.348	-.766**	0.497
Sphingolipid metabolism	-0.391	.704*	-0.028	-0.41	-0.265	.861**	-0.27	-0.306	.750**	0.415	-0.208	0.049
ABC transporters	-0.528	.722**	.613*	-0.041	-0.49	0.023	-0.383	0.055	.677*	.827**	-0.328	-0.311
Phosphotransferase system (PTS)	-0.305	0.476	-0.251	-0.403	0.107	0.453	-0.012	-0.145	0.435	0.453	0.141	-0.05

Bacterial secretion system	-0.459	0.072	-0.183	0.133	.763**	0.009	.778**	.584*	-0.305	-0.429	.741**	-0.107
Riboflavin metabolism	-.620*	0.127	0.251	0.369	.728**	-0.403	.716**	.917**	-0.348	-0.314	.754**	-0.41
Vitamin B6 metabolism	.747**	-.909**	-0.512	0.346	0.133	-0.194	0.166	-0.026	-.579*	-.734**	0.247	0.051
Nicotinate and nicotinamide metabolism	.614*	-.863**	-0.293	0.487	0.15	-0.475	0.26	0.271	-.606*	-.704*	0.366	-0.219
Porphyrin and chlorophyll metabolism	-.611*	.932**	0.448	-0.367	-0.33	0.4	-0.449	-0.072	.767**	.819**	-0.375	-0.238
Phosphonate and phosphinate metabolism	-.660*	.944**	.619*	-0.427	-0.272	0.387	-0.441	-0.114	.663*	.629*	-0.519	-0.004
Purine metabolism	-0.035	-0.536	-0.224	0.543	.721**	-0.434	.815**	.646*	-.622*	-.681*	.893**	-0.174
Pyrimidine metabolism	0.37	-.751**	-0.54	0.393	0.57	-0.228	.600*	0.321	-.676*	-.825**	.654*	-0.007
Two-component system	-.612*	.900**	0.171	-0.469	-0.136	0.539	-0.228	-0.083	.689*	.670*	-0.173	-0.122
mRNA surveillance pathway	-0.011	0.192	0.086	-0.079	-0.146	0.077	-0.387	-0.305	0.138	0.344	-.601*	0.302
Peroxisome	-0.447	.852**	0.515	-0.516	-0.44	0.407	-.631*	-0.338	.735**	.783**	-.625*	-0.003
Aminobenzoate degradation	-0.412	.718**	.707*	-0.341	-0.506	0.082	-0.57	-0.201	.590*	.729**	-.602*	-0.056

A value greater than zero represents a positive correlation, a value less than zero represents a negative correlation. The significant correlations are presented as asterisks (*, $p < 0.05$;**, $p < 0.01$).

Table S10. The Pearson's correlation analysis between soil bacterial genera and key metabolic pathways related to the C cycle

	RB41	Sphingomonas	H16	Acidobacteria_bacterium_WX27	Skermanella	Lysobacter	Steroidobacter	Acidibacter	Gaiella	Bacillus	AKYG587	Nocardioides	Nitrospira	Pseudarthrobacter	CandidatusEntotheonella	Haliangium	Streptomyces	DS-100
Glycine, serine and threonine metabolism	0.524	-0.522	-0.077	0.413	-0.284	-0.128	-0.318	-0.354	-0.228	-0.412	-0.186	-0.258	-0.37	0.018	-0.348	0.076	-0.385	0.419
Cysteine and methionine metabolism	.784**	-0.148	-0.141	0.381	-0.275	-0.057	-0.058	-0.245	-.654*	-0.513	0.143	-0.545	-0.463	-0.089	-0.357	-0.244	-.764**	0.303
Valine, leucine and isoleucine biosynthesis	-0.07	-.664*	0.524	-0.148	-.611*	0.367	-.684*	-0.472	0.146	-0.451	0.421	-0.238	-0.14	-0.544	-0.495	0.531	-0.16	-0.194
Arginine and proline metabolism	-0.032	-0.312	-0.513	0.553	.760**	-.660*	.623*	0.491	0.034	.582*	-.842**	0.063	0.296	0.421	.604*	-0.248	0.241	0.351
Tryptophan metabolism	-0.252	0.232	-0.523	0.339	.747**	-0.471	.663*	0.525	0.279	.689*	-.790**	0.472	0.353	0.516	.593*	-0.383	0.522	0.214
Glycolysis / Gluconeogenesis	-.634*	0.118	0.29	-0.53	0.336	0.133	0.066	0.408	0.543	.580*	0.009	0.429	.641*	0.045	0.503	0.106	.724**	-.583*
Citrate cycle (TCA cycle)	-0.01	0.337	0.322	-0.224	-.839**	0.565	-.657*	-.658*	0.182	-0.555	0.528	0.254	-0.401	-0.348	-.838**	0.108	-0.049	-0.027
Pentose phosphate pathway	0.559	-0.546	-0.205	0.453	-0.339	-0.115	-0.214	-0.418	-0.427	-0.47	0.039	-0.238	-0.515	-0.341	-0.431	-0.088	-0.538	0.097

Pentose and glucuronate interconversions	0.301	0.165	-.816**	.723**	.699*	-.633*	.845**	0.456	-0.291	0.407	-.757**	0.058	-0.014	0.43	0.445	-.650*	-0.112	0.376
Fructose and mannose metabolism	.728**	-0.155	-0.524	.664*	-0.196	-0.374	-0.012	-0.432	-0.569	-0.472	-0.201	-0.184	-.670*	0.06	-0.435	-0.268	-0.557	.633*
Galactose metabolism	.856**	0.219	-.710**	.598*	-0.032	-0.485	0.15	-0.331	-.687*	-0.323	-0.191	0.054	-.636*	0.21	-0.306	-.604*	-0.423	0.531
Ascorbate and aldarate metabolism	-0.492	-0.066	-0.1	-0.026	.813**	-0.292	.599*	.761**	0.346	.825**	-0.548	0.15	.704*	0.345	.864**	-0.073	0.541	-0.17
Starch and sucrose metabolism	0.133	0.287	-0.338	-0.039	.641*	-0.407	0.497	0.555	-0.146	0.552	-0.348	0.203	0.321	0.458	.640*	-0.441	0.239	-0.125
Amino sugar and nucleotide sugar metabolism	.853**	-0.058	-0.426	0.505	-0.273	-0.298	-0.094	-0.452	-.688*	-0.542	-0.003	-0.238	-.676*	0.038	-0.465	-0.316	-.619*	0.53
Inositol phosphate metabolism	-0.276	0.228	-0.456	0.102	.658*	-0.49	0.461	0.414	0.269	.695*	-.586*	.631*	0.343	0.421	0.551	-0.322	.657*	-0.017
Pyruvate metabolism	-.610*	-0.143	0.262	-0.378	.607*	-0.039	0.317	.685*	0.45	.783**	-0.21	0.177	.839**	0.129	.781**	0.056	.674*	-0.467
Glyoxylate and dicarboxylate metabolism	-0.411	-0.233	-0.24	0.355	.677*	-0.381	0.529	0.568	0.422	.676*	-.790**	0.152	0.501	0.358	.590*	-0.055	0.436	0.205
Propanoate metabolism	0.219	-0.005	-.734**	.797**	0.546	-.590*	.654*	0.314	-0.073	0.426	-.916**	0.28	0.012	0.421	0.271	-.653*	0.132	0.567
Butanoate metabolism	-0.514	0.118	-0.273	0.099	.653*	-0.322	0.487	0.513	0.491	.750**	-.653*	0.541	0.502	0.376	.587*	-0.177	.710**	0.019
C5-Branched dibasic acid metabolism	-0.34	-.640*	.689*	-0.358	-0.547	0.469	-.680*	-0.345	0.335	-0.258	0.487	-0.138	0.095	-.604*	-0.359	.592*	0.077	-0.418
Methane metabolism	-.708*	-0.111	0.195	-0.271	.617*	-0.037	0.355	.662*	0.558	.770**	-0.31	0.203	.795**	0.152	.751**	0.127	.668*	-0.4
Carbon fixation in photosynthetic organisms	0.198	-0.146	.601*	-0.445	-.785**	.581*	-.735**	-0.53	-0.161	-.697*	.831**	-0.376	-0.312	-0.498	-.598*	0.416	-0.443	-0.308
Carbon fixation pathways in prokaryotes	.580*	-0.14	-0.004	0.279	-.653*	0.138	-0.425	-.621*	-0.414	-.691*	0.287	-0.263	-.623*	-0.277	-.743**	-0.065	-0.573	0.331
ABC transporters	-0.552	-0.289	-0.048	-0.006	.722**	-0.319	0.43	.623*	0.446	.787**	-0.529	0.225	.689*	0.226	.757**	0.043	.634*	-0.195
One carbon pool by folate	-0.248	0.091	.752**	-.719**	-.692*	.788**	-.622*	-0.321	0.143	-0.421	.867**	-0.158	-0.038	-0.536	-0.421	0.447	-0.125	-0.575
Cyanoamino acid metabolism	0.028	0.014	-.683*	.722**	0.555	-.596*	0.528	0.239	0.146	0.361	-.877**	0.283	0.01	0.479	0.27	-0.306	0.182	0.541

A value greater than zero represents a positive correlation, a value less than zero represents a negative correlation. The significant correlations are presented as asterisks (*, $p < 0.05$;**, $p < 0.01$).

Table S11. The Pearson's correlation analysis between soil bacterial genera and key metabolic pathways related to the N cycle

	RB41	Sphingomonas	H16	Acidobacteria_bacterium_WX27	Skermanella	Lysobacter	Steroidobacter	Acidibacter	Gaiella	Bacillus	AKYG587	Nocardioides	Nitrospira	Pseudarthrobacter	CandidatusEntotheonella	Haliangium	Streptomyces	DS-100
Alanine, aspartate and glutamate metabolism	.324	-.260	.503	-.336	-.738**	.464	-.709**	-.562	-.276	-.652*	.702*	-.323	-.331	-.452	-.563	.279	-.381	-.205
Glycine, serine and threonine metabolism	.524	-.522	-.077	.413	-.284	-.128	-.318	-.354	-.228	-.412	-.186	-.258	-.370	.018	-.348	.076	-.385	.419

Cysteine and methionine metabolism	.784**	-.148	-.141	.381	-.275	-.057	-.058	-.245	-.654*	-.513	.143	-.545	-.463	-.089	-.357	-.244	-.764**	.303
Arginine and proline metabolism	-.032	-.312	-.513	.553	.760**	-.660*	.623*	.491	.034	.582*	-.842**	.063	.296	.421	.604*	-.248	.241	.351
Histidine metabolism	.634*	-.342	-.193	.531	-.413	-.037	-.169	-.421	-.482	-.542	-.007	-.366	-.573	-.214	-.528	-.154	-.663*	.365
Tyrosine metabolism	.146	.389	-.702*	.494	.766**	-.612*	.789**	.523	-.098	.511	-.764**	.204	.143	.661*	.543	-.552	.146	.467
Phenylalanine metabolism	-.249	.178	-.468	.294	.799**	-.538	.624*	.551	.286	.679*	-.767**	.358	.403	.593*	.650*	-.247	.504	.269
Tryptophan metabolism	-.252	.232	-.523	.339	.747**	-.471	.663*	.525	.279	.689*	-.790**	.472	.353	.516	.593*	-.383	.522	.214
Pentose phosphate pathway	.559	-.546	-.205	.453	-.339	-.115	-.214	-.418	-.427	-.470	.039	-.238	-.515	-.341	-.431	-.088	-.538	.097
C5-Branched dibasic acid metabolism	-.340	-.640*	.689*	-.358	-.547	.469	-.680*	-.345	.335	-.258	.487	-.138	.095	-.604*	-.359	.592*	.077	-.418
Nitrogen metabolism	.433	-.092	-.610*	.738**	.666*	-.570	.781**	.527	-.371	.390	-.751**	-.224	.113	.416	.478	-.636*	-.216	.435
One carbon pool by folate	-.248	.091	.752**	-.719**	-.692*	.788**	-.622*	-.321	.143	-.421	.867**	-.158	-.038	-.536	-.421	.447	-.125	-.575
Nicotinate and nicotinamide metabolism	.340	-.365	.033	.298	-.712**	.185	-.561	-.732**	-.159	-.657*	.196	-.075	-.605*	-.403	-.784**	.124	-.377	.215
Taurine and hypotaurine metabolism	.680*	.012	-.595*	.732**	.329	-.512	.436	.135	-.454	-.068	-.591*	-.290	-.264	.497	.099	-.423	-.458	.724**
Selenocompound metabolism	.774**	-.010	-.524	.657*	-.227	-.328	-.013	-.424	-.556	-.504	-.182	-.172	-.690*	.113	-.481	-.319	-.595*	.658*
Cyanoamino acid metabolism	.028	.014	-.683*	.722**	.555	-.596*	.528	.239	.146	.361	-.877**	.283	.010	.479	.270	-.306	.182	.541
D-Glutamine and D-glutamate metabolism	.037	.056	.645*	-.642*	-.805**	.620*	-.801**	-.542	-.028	-.562	.848**	-.091	-.231	-.434	-.590*	.375	-.148	-.350
Purine metabolism	.081	-.244	.590*	-.454	-.792**	.578*	-.766**	-.603*	-.115	-.620*	.798**	-.218	-.287	-.589*	-.586*	.405	-.254	-.380
Two-component system	-.486	.101	.215	-.293	.645*	.036	.553	.832**	.238	.715**	-.205	-.152	.772**	.224	.819**	-.034	.329	-.323
Chloroalkane and chloroalkene degradation	-.336	.026	-.338	.236	.856**	-.416	.703*	.714**	.307	.767**	-.738**	.228	.557	.467	.792**	-.230	.468	.053
Aminobenzoate degradation	-.453	.105	-.312	.095	.676*	-.401	.458	.465	.429	.689*	-.654*	.492	.442	.455	.604*	-.096	.669*	.099
Atrazine degradation	-.421	-.189	-.186	.062	.784**	-.462	.476	.580*	.324	.706*	-.580*	.169	.570	.397	.768**	.060	.543	.015

A value greater than zero represents a positive correlation, a value less than zero represents a negative correlation. The significant correlations are presented as asterisks (*, $p < 0.05$;**, $p < 0.01$).

Table S12. The Pearson's correlation analysis between soil bacterial genera and key metabolic pathways related to the P cycle

	RB41	Sphingomonas	H16	Acidobacteria_bacterium_WX27	Skermanella	Lysobacter	Steroidobacter	Acidibacter	Gaiella	Bacillus	AKYG587	Nocardioides	Nitrospira	Pseudarthrobacter	CandidatusEntotheonella	Haliangium	Streptomyces	DS-100
Glycine, serine and threonine metabolism	.524	-.522	-.077	.413	-.284	-.128	-.318	-.354	-.228	-.412	-.186	-.258	-.370	.018	-.348	.076	-.385	.419
Cysteine and methionine metabolism	.784**	-.148	-.141	.381	-.275	-.057	-.058	-.245	-.654*	-.513	.143	-.545	-.463	-.089	-.357	-.244	-.764**	.303
Histidine metabolism	.634*	-.342	-.193	.531	-.413	-.037	-.169	-.421	-.482	-.542	-.007	-.366	-.573	-.214	-.528	-.154	-.663*	.365
Streptomycin biosynthesis	.442	-.303	.265	-.115	-.751**	.269	-.715**	-.706*	-.337	-.742**	.569	-.247	-.544	-.409	-.684*	.261	-.455	-.026
Glycolysis / Gluconeogenesis	-.634*	.118	.290	-.530	.336	.133	.066	.408	.543	.580*	.009	.429	.641*	.045	.503	.106	.724**	-.583*
Fructose and mannose metabolism	.728**	-.155	-.524	.664*	-.196	-.374	-.012	-.432	-.569	-.472	-.201	-.184	-.670*	.060	-.435	-.268	-.557	.633*
Galactose metabolism	.856**	.219	-.710**	.598*	-.032	-.485	.150	-.331	-.687*	-.323	-.191	.054	-.636*	.210	-.306	-.604*	-.423	.531
Starch and sucrose metabolism	.133	.287	-.338	-.039	.641*	-.407	.497	.555	-.146	.552	-.348	.203	.321	.458	.640*	-.441	.239	-.125
Amino sugar and nucleotide sugar metabolism	.853**	-.058	-.426	.505	-.273	-.298	-.094	-.452	-.688*	-.542	-.003	-.238	-.676*	.038	-.465	-.316	-.619*	.530
Inositol phosphate metabolism	-.276	.228	-.456	.102	.658*	-.490	.461	.414	.269	.695*	-.586*	.631*	.343	.421	.551	-.322	.657*	-.017
Pyruvate metabolism	-.610*	-.143	.262	-.378	.607*	-.039	.317	.685*	.450	.783**	-.210	.177	.839**	.129	.781**	.056	.674*	-.467
Glyoxylate and dicarboxylate metabolism	-.411	-.233	-.240	.355	.677*	-.381	.529	.568	.422	.676*	-.790**	.152	.501	.358	.590*	-.055	.436	.205
Oxidative phosphorylation	.158	-.119	.439	-.001	-.790**	.641*	-.462	-.418	-.078	-.616*	.498	-.417	-.347	-.541	-.717**	.155	-.581*	-.077
Methane metabolism	-.708*	-.111	.195	-.271	.617*	-.037	.355	.662*	.558	.770**	-.310	.203	.795**	.152	.751**	.127	.668*	-.400
Carbon fixation in photosynthetic organisms	.198	-.146	.601*	-.445	-.785**	.581*	-.735**	-.530	-.161	-.697*	.831**	-.376	-.312	-.498	-.598*	.416	-.443	-.308
RNA degradation	-.360	.060	.555	-.324	-.623*	.802**	-.237	-.135	.124	-.302	.632*	-.304	-.076	-.709**	-.445	.239	-.359	-.506
Lipopolysaccharide biosynthesis	.575	-.299	.143	.181	-.470	.176	-.268	-.364	-.535	-.627*	.359	-.643*	-.431	-.289	-.453	.049	-.743**	.183
Glycerolipid metabolism	.038	.051	-.359	.086	.662*	-.306	.683*	.605*	-.244	.604*	-.381	.119	.337	.182	.706*	-.541	.180	-.266
Glycerophospholipid metabolism	.284	-.508	.423	.064	-.496	.488	-.305	-.187	-.210	-.421	.232	-.545	-.136	-.410	-.327	.090	-.514	-.080
Arachidonic acid metabolism	.498	.615*	-.652*	.439	.535	-.516	.563	.277	-.276	.179	-.490	.142	-.064	.697*	.284	-.623*	-.047	.527
Sphingolipid metabolism	-.393	.079	.063	.001	.460	.232	.696*	.788**	.162	.566	-.193	-.160	.539	-.136	.571	-.301	-.003	-.509
ABC transporters	-.552	-.289	-.048	-.006	.722**	-.319	.430	.623*	.446	.787**	-.529	.225	.689*	.226	.757**	.043	.634*	-.195

Phosphotransferase system (PTS)	-.024	.166	.172	-.350	.477	-.048	.391	.554	-.280	.415	.056	-.361	.489	.263	.673*	-.119	.079	-.164
Bacterial secretion system	-.272	.031	.783**	-.711**	-.520	.755**	-.447	-.138	.076	-.323	.850**	-.353	.105	-.514	-.225	.442	-.176	-.590*
Riboflavin metabolism	-.512	-.259	.919**	-.735**	-.535	.731**	-.693*	-.205	.465	-.191	.661*	-.110	.277	-.477	-.220	.667*	.201	-.591*
Vitamin B6 metabolism	.564	-.156	-.091	.376	-.671*	.179	-.384	-.600*	-.324	-.669*	.185	-.119	-.664*	-.343	-.783**	-.159	-.575	.232
Nicotinate and nicotinamide metabolism	.340	-.365	.033	.298	-.712**	.185	-.561	-.732**	-.159	-.657*	.196	-.075	-.605*	-.403	-.784**	.124	-.377	.215
Porphyrin and chlorophyll metabolism	-.570	-.042	.093	-.092	.750**	-.095	.566	.835**	.476	.839**	-.478	.087	.837**	.282	.841**	-.067	.546	-.261
Phosphonate and phosphinate metabolism	-.654*	.251	-.035	-.079	.594*	-.067	.478	.615*	.585*	.719**	-.419	.353	.626*	.267	.595*	-.061	.607*	-.201
Purine metabolism	.081	-.244	.590*	-.454	-.792**	.578*	-.766**	-.603*	-.115	-.620*	.798**	-.218	-.287	-.589*	-.586*	.405	-.254	-.380
Pyrimidine metabolism	.369	-.108	.345	-.121	-.817**	.478	-.612*	-.623*	-.303	-.743**	.655*	-.318	-.505	-.502	-.743**	.155	-.547	-.093
Two-component system	-.486	.101	.215	-.293	.645*	.036	.553	.832**	.238	.715**	-.205	-.152	.772**	.224	.819**	-.034	.329	-.323
mRNA surveillance pathway	.138	.432	-.372	.222	.461	-.584*	.249	.183	.077	.305	-.252	.256	.176	.518	.174	-.309	.304	.367
Peroxisome	-.415	.254	-.262	.080	.774**	-.318	.639*	.678*	.370	.792**	-.626*	.367	.587*	.477	.724**	-.278	.600*	.022
Aminobenzoate degradation	-.453	.105	-.312	.095	.676*	-.401	.458	.465	.429	.689*	-.654*	.492	.442	.455	.604*	-.096	.669*	.099

A value greater than zero represents a positive correlation, a value less than zero represents a negative correlation. The significant correlations are presented as asterisks (*, $p < 0.05$;**, $p < 0.01$).

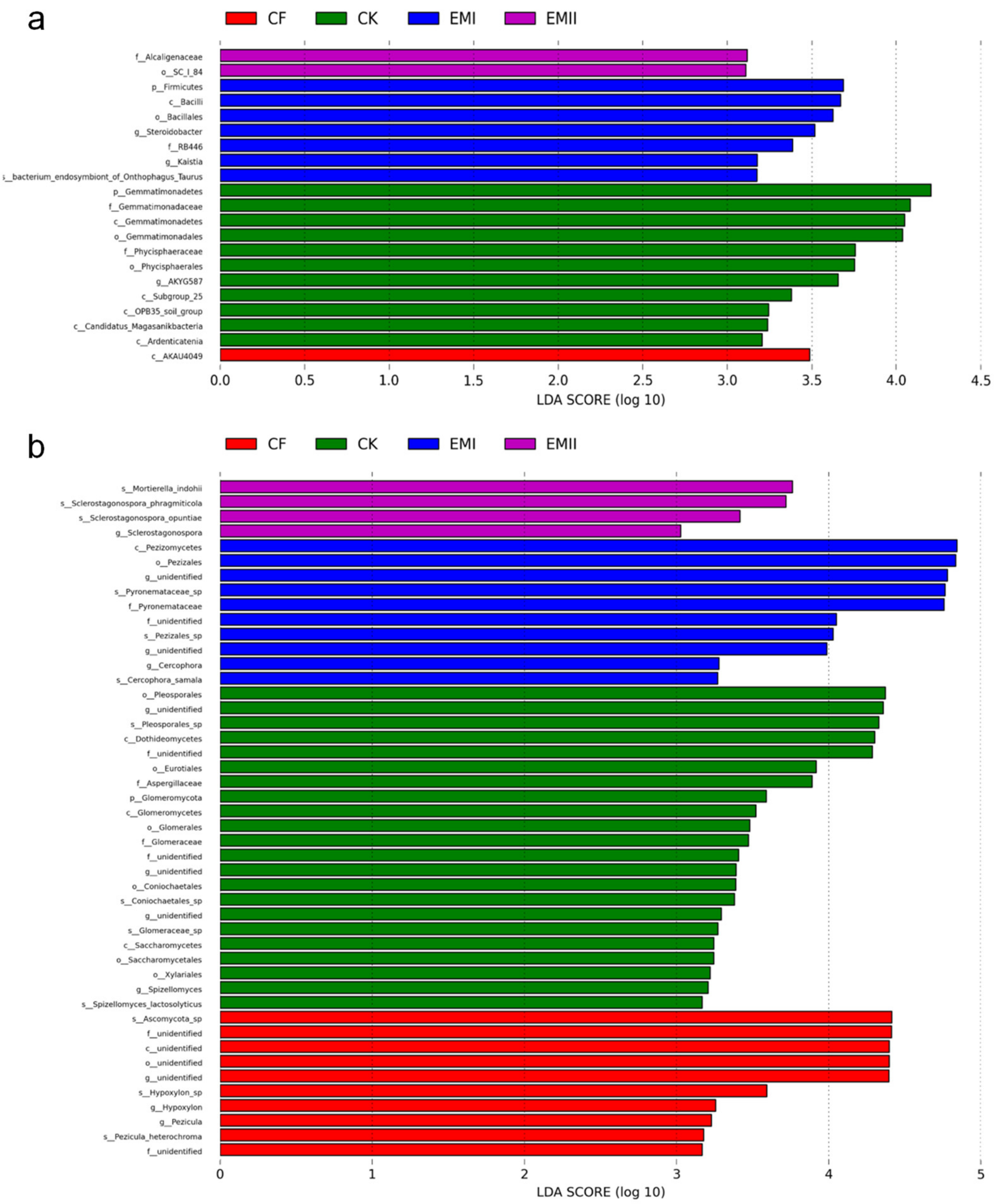

Figure S1. The linear discriminant analysis (LDA) effect size (LEfSe) of soil bacterial (a) and fungal (b) biomarkers under different fertilization treatments. Identified phylotype biomarkers were ranked by effect size (> 3), and the α value was < 0.05.

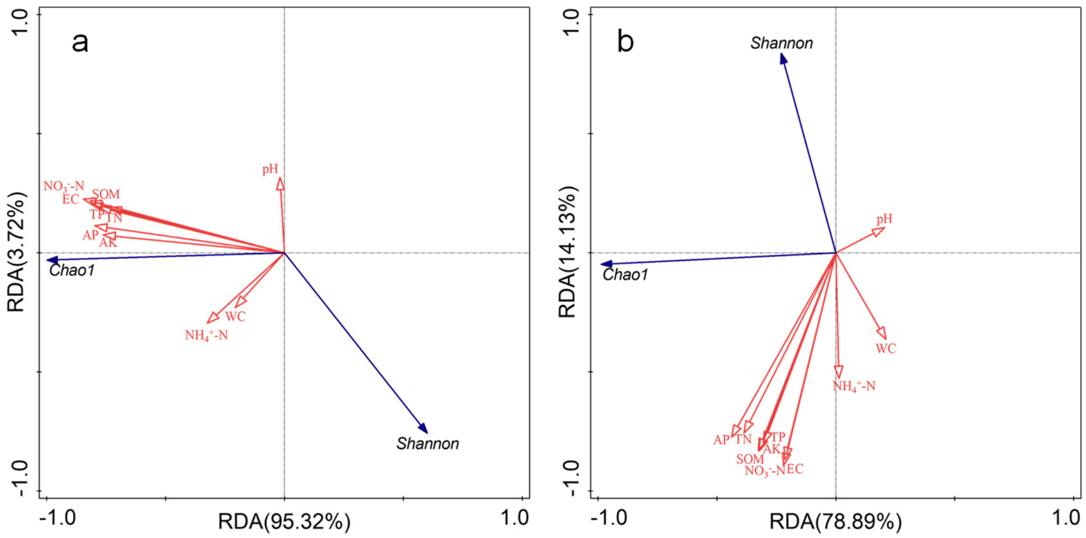

Figure S2. Redundancy analysis (RDA) between soil bacterial (a) and fungal (b) alpha-diversity and soil properties under different fertilization treatments. Red arrows represent soil properties; blue arrows represent alpha-diversity.

Spearman's correlation analysis

Spearman's correlation analysis was used to evaluate the relationship between soil properties and the relative abundance of soil bacteria and fungi at the phylum and genus level (Figures. S3 and S4). The relative abundances of bacterial dominant taxa had a good correlation with soil properties. For instance, soil EC, SOM, TP, TN, NO_3^--N, AP, and AK were positively correlated with the relative abundance of Firmicutes, Bacteroidetes, and Tectomicrobia, and negatively correlated with that of Gemmatimonadetes, Planctomycetes, Nitrospirae, and Latescibacteria (Figure S3a). However, the fungal dominant phyla of were only significantly correlated with a few soil properties. The relative abundance of Ascomycota was positively correlated with soil TP, TN, and AP, and that of Mortierellomycota was negatively correlated with soil pH (Figure S3b). Similarly, at the genus level, soil EC, SOM, TP, TN, NO_3^--N, AP, and AK had a positive correlation with Skermanella, Steroidobacter, Acidobacteria_bacterium_WX27 genus, and Bacillus, and a negative correlation with that of H16, AKYG587, Lysobacter, and Halianhium (Figure S4a). The relative abundances of Gaiella and Nocardioides within Actinobacteria was significantly positively correlated with pH and NH_4^+-N, respectively. In addition, the relative abundance of Chrysosporium showed a significantly positive relationship with EC, the relative abundance of Mortierella was significantly negatively correlated with pH; the relative abundance of Stachybotrys, Fusicolla, and Xeromyces was significantly negatively correlated with SOM and AK (Figure S4b). Therefore, bacteria were more sensitive to changes in soil properties than fungi.

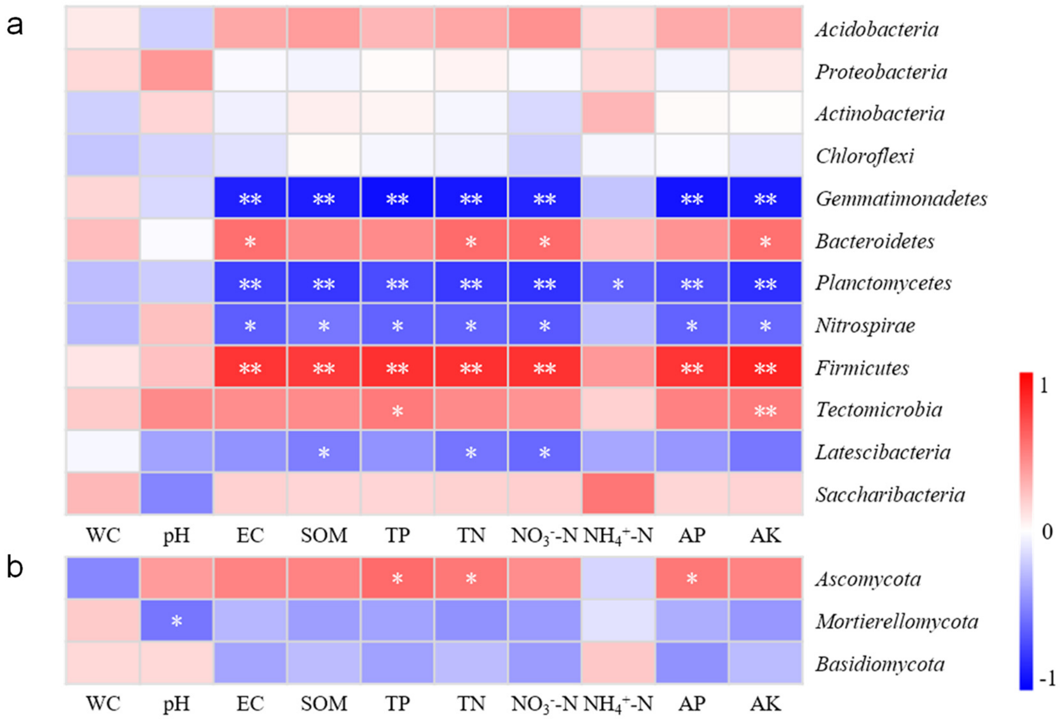

Figure S3. The heatmap of the correlation between soil properties and the relative abundance of bacterial (a) and fungal (b) phylum under different fertilization treatments (relative abundance > 1%). This heatmap was created according to the result of Spearman's correlation analysis. Positive relationships are represented in red, while negative relationships are represented in blue. The significant correlations are presented as asterisks (*, $p < 0.05$;**, $p < 0.01$).

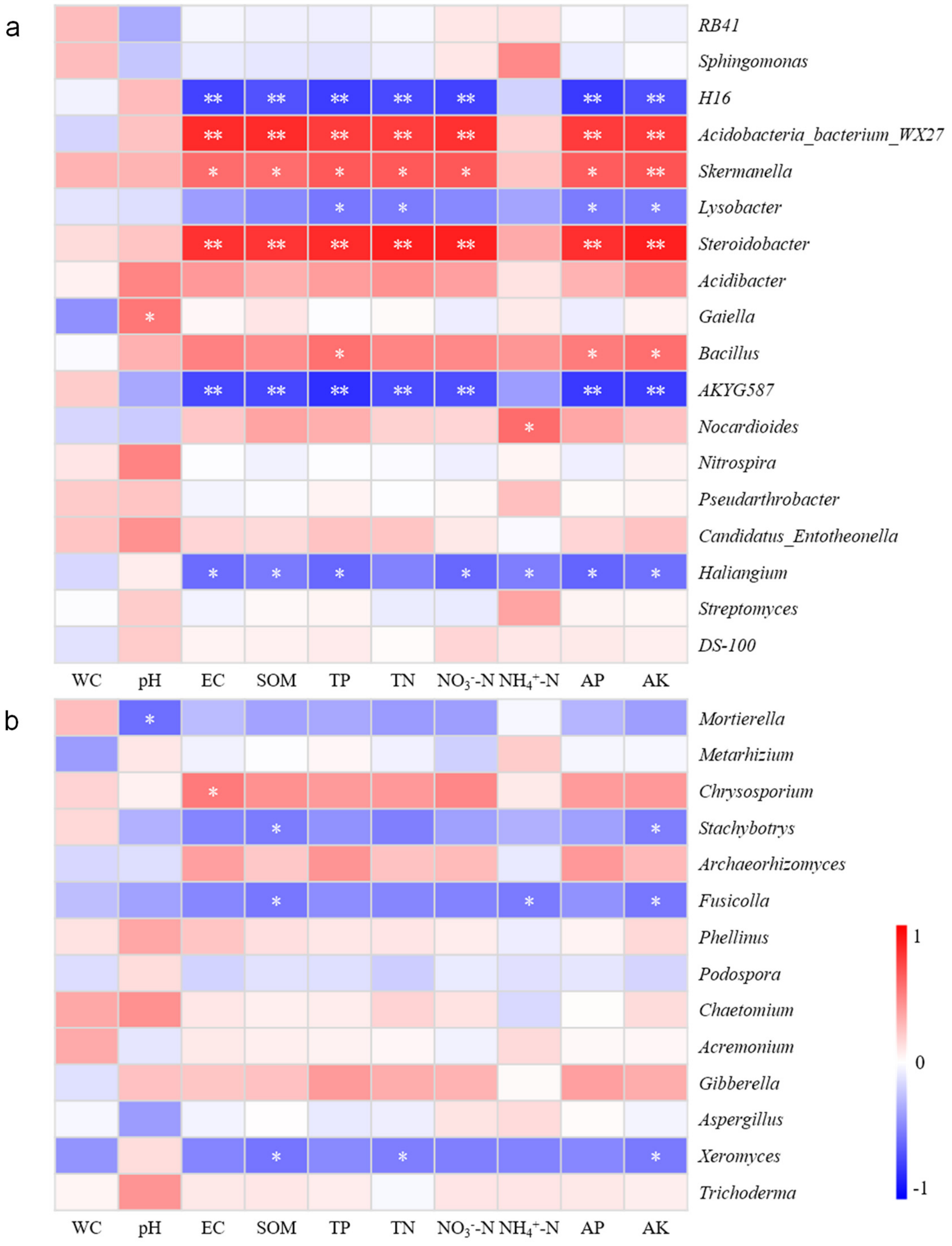

Figure S4. The heatmap of the correlation between soil properties and the relative abundance of bacterial (a) and fungal (b) genera under different fertilization treatments (relative abundance > 1%). This heatmap was created according to the result of Spearman's correlation analysis. Positive relationships are represented in red, while negative relationships are represented in blue. The significant correlations are presented as asterisks (*, $p < 0.05$;**, $p < 0.01$).

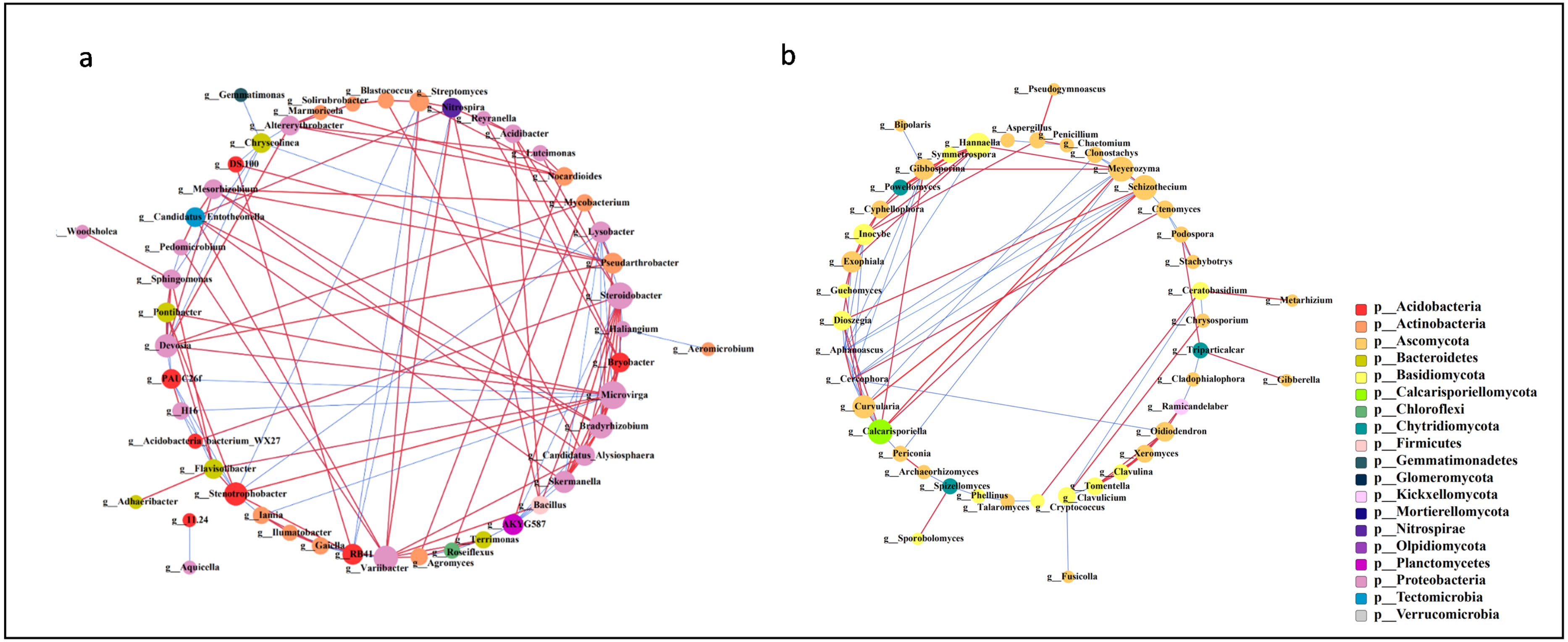

Figure S5. Network analysis showing overall bacterial (a) and fungal (b) co-occurrence. The co-occurring networks are colored by microbial taxonomic information at the phylum level. The red lines represent significantly positive (r > 0.6) interrelationships, blue lines represent negative (r < -0.6) interrelationships. The size of the node represents the abundance, and the width of the lines represents the strength of the correlation.

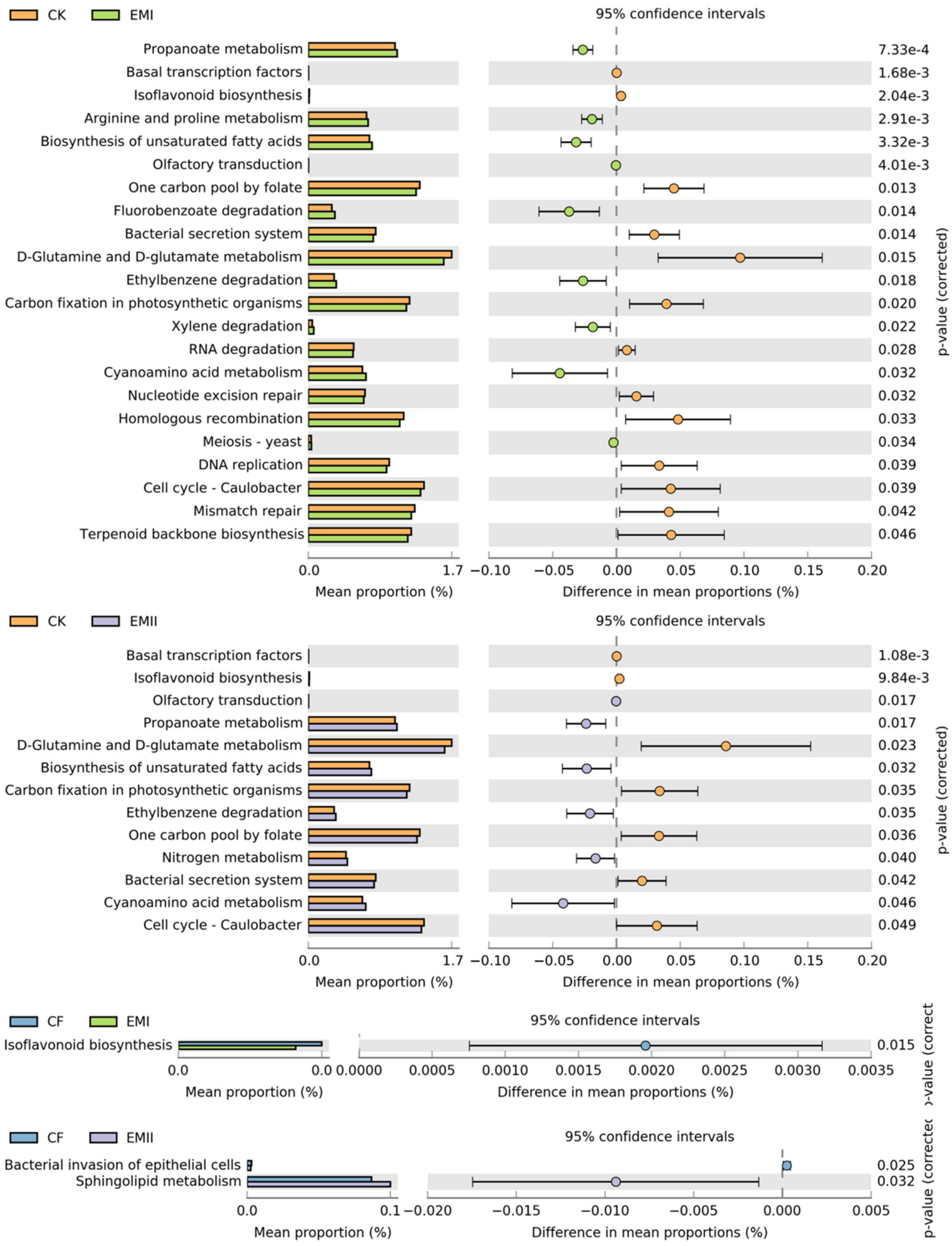

Figure S6. Extended error bar plots showing statistically significant differences in the bacterial functional groups between the fertilization treatments and the control treatment. Error bars indicate within-group standard deviations. Presented categories passed a corrected P value of < 0.05 in Welch's t test.

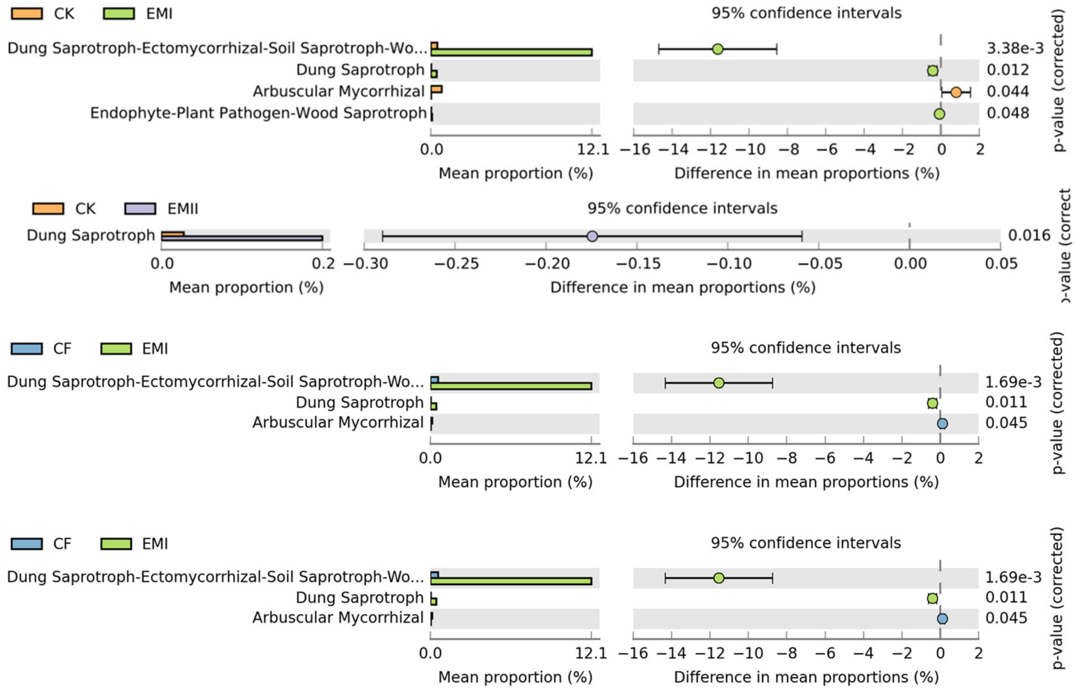

Figure S7. Extended error bar plots showing statistically significant differences in the fungal functional groups (guilds) between the fertilization treatments and the control treatment. Error bars indicate within-group standard deviations. Presented categories passed a corrected P value of < 0.05 in Welch's t test.
